# Supplementary material for: Indoor running temporal variability for different running speeds, treadmill inclinations, and three different estimation strategies
Source: PLoS One. 2023 Jul 20;18(7):e0287978. doi: 10.1371/journal.pone.0287978 (PMC10358961; doi:10.1371/journal.pone.0287978)
Supplement: S1 File — (PDF) [file pone.0287978.s001.pdf]

Additional material - Indoor running temporal variability for different running speeds, treadmill inclinations, and three different estimation strategies

Complete dataset (20 participants)

Results are given here for the entire sample of participants (n=20). Results are provided for stride frequency, duty factor, DFA-alpha and Higuchi's D. The data presented in this section were collected with the Optogait optical system. Data were analysed with ANOVA (parametric/non-parametric) test after checking for normality. ANOVA conditions were running speed (i.e.: 80, 90, 100, 110, or 120% of PRS) and treadmill inclination (i.e.: -8, -5, -2, 2, 5, and 8% of inclination).

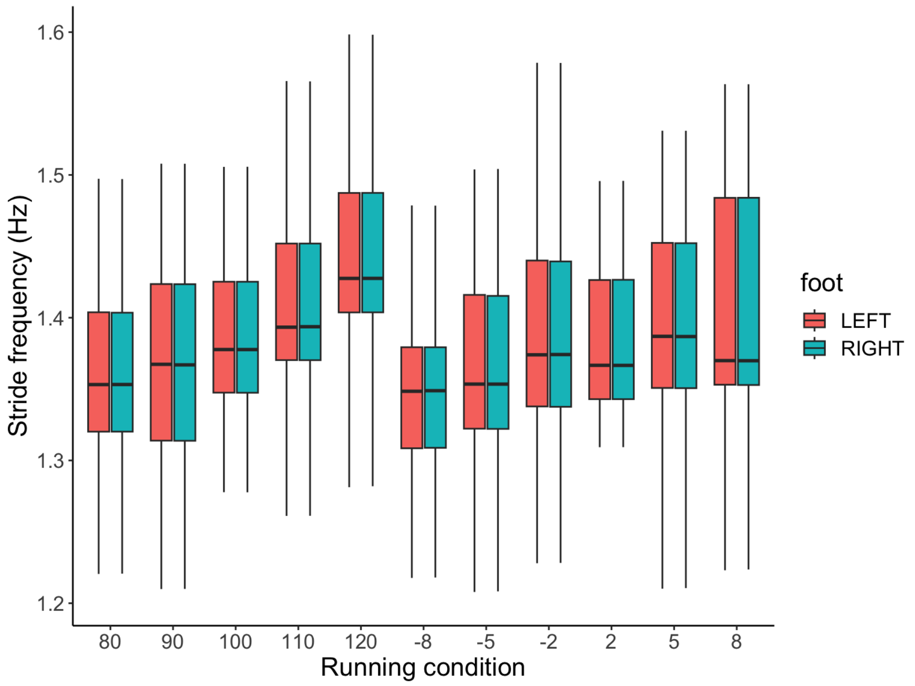

Stride frequency right and left foot obtained with Optogait (Opto) for all the participants together in different running conditions.

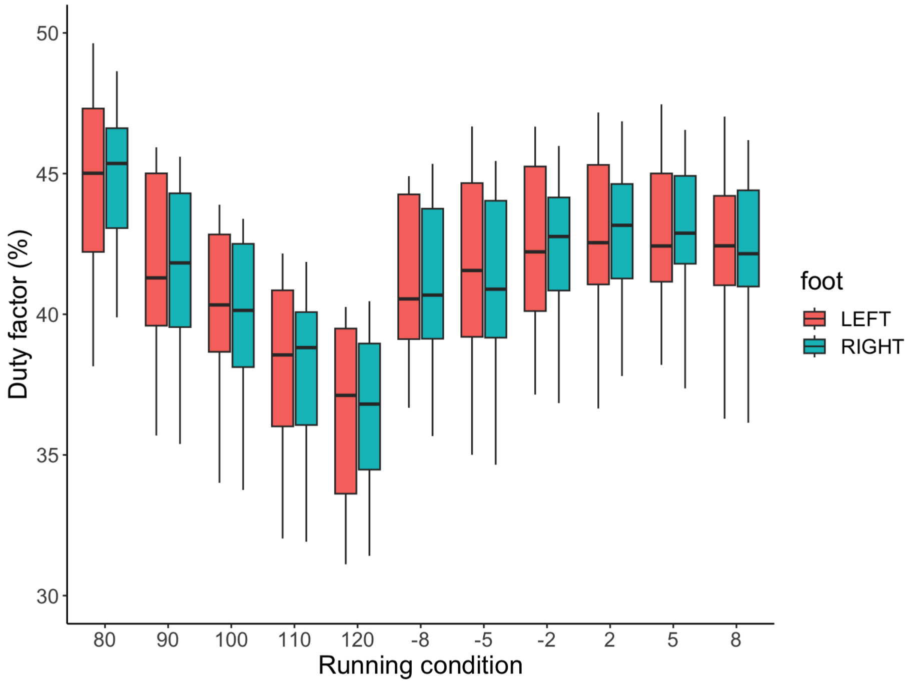

Duty factor right and left foot obtained with Optogait (Opto) for all the participants together in different running conditions.

```
## Joining, by = c("SBJ", "CND")
```

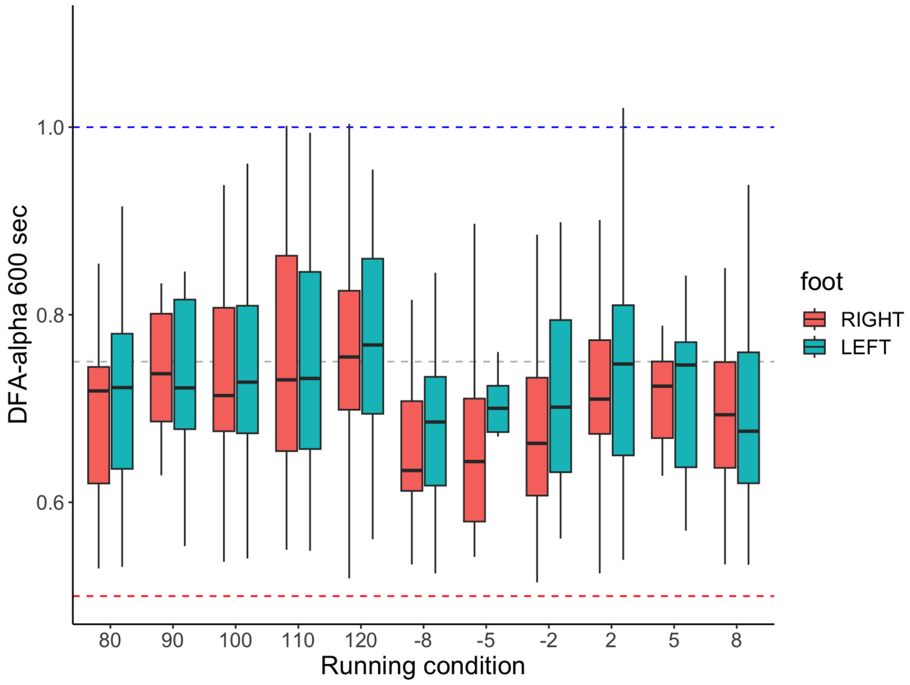

DFA-alpha computed from stride frequency right foot obtained with Optogait (Opto) for all the participants together in different running conditions. DFA-alpha has been computed using the entire duration of the test (600 sec). Horizontal red and blue dashed lines indicate an uncorrelated random signal and a signal with strong long-term correlations, respectively.

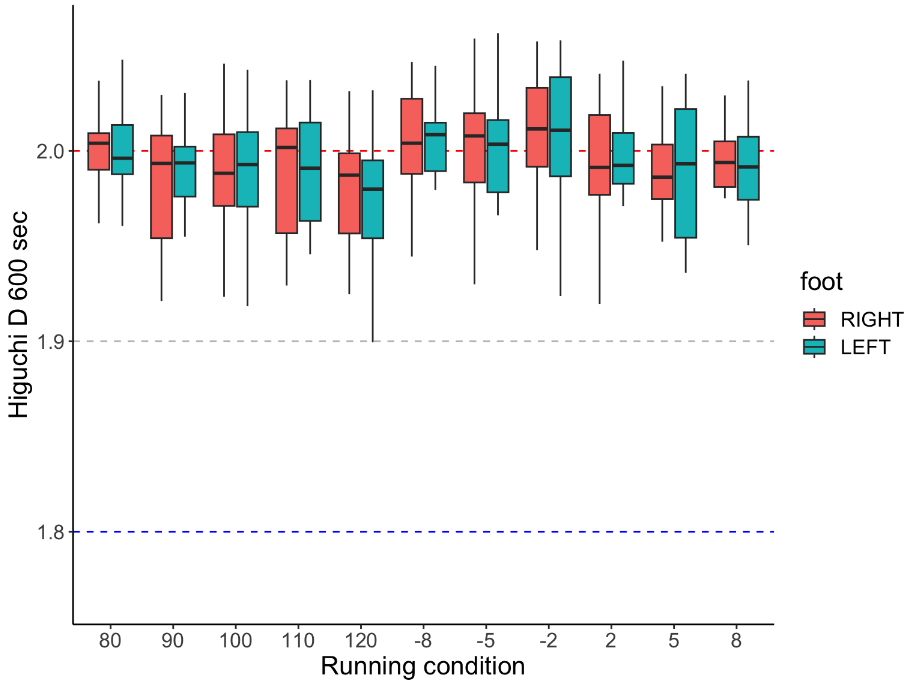

Higuchi’s D computed from stride frequency right foot obtained with Optogait (Opto) for all the participants together in different running conditions. Higuchi’s D has been computed using the entire duration of the test (600 sec). Horizontal red and blue dashed lines indicate an uncorrelated random signal and a signal with strong long-term correlations, respectively.

Influence of speed on running frequency

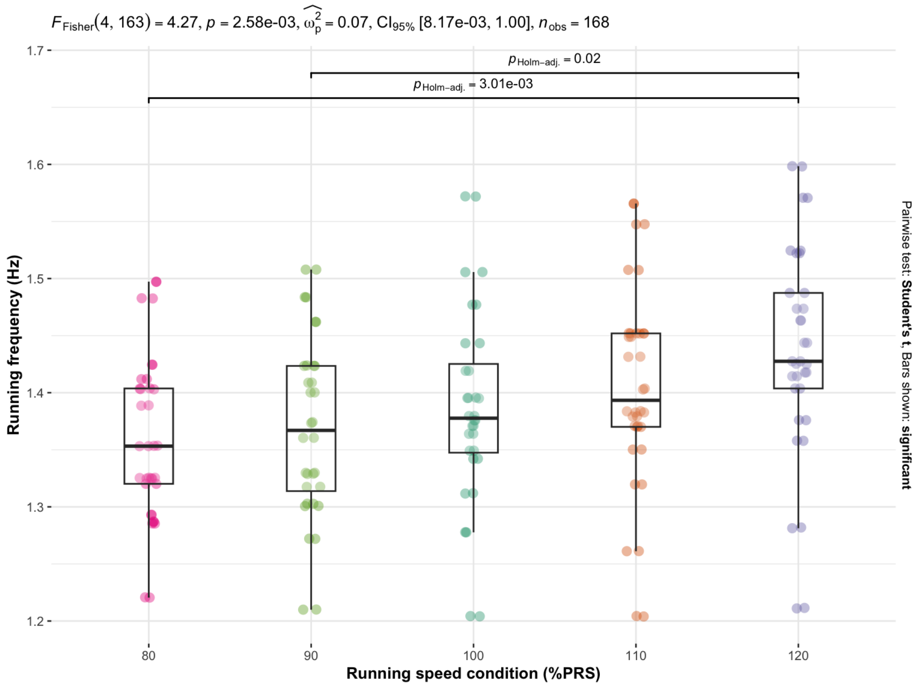

ANOVA and post-hoc tests analysis for running frequency with different running speeds.

Influence of speed on duty factor

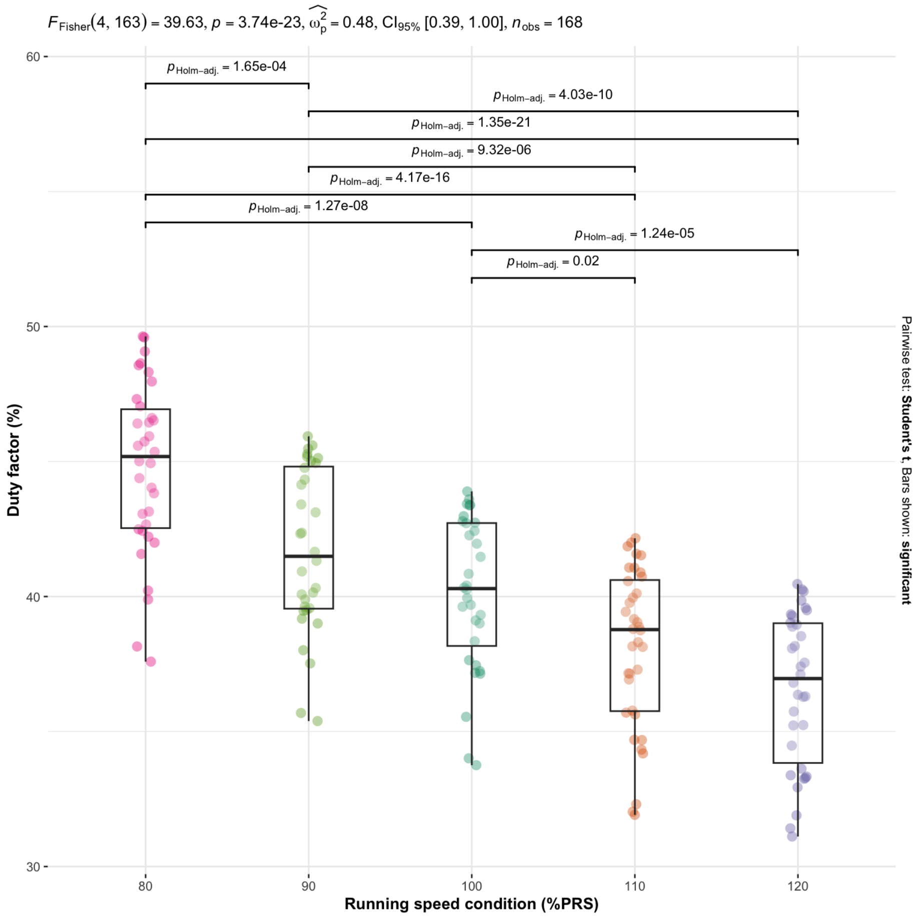

ANOVA and post-hoc tests analysis for duty factor with different running speeds.

Influence of speed on DFA-alpha

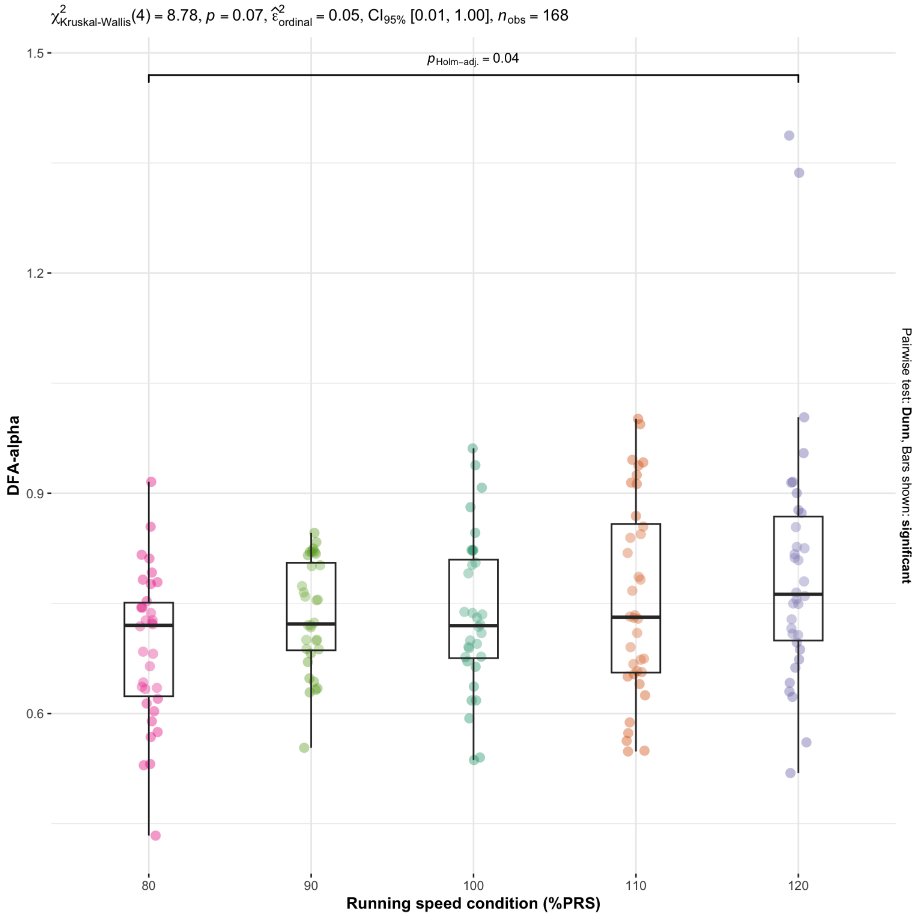

ANOVA and post-hoc tests analysis for DFA-alpha with different running speeds.

Influence of speed on HG-D

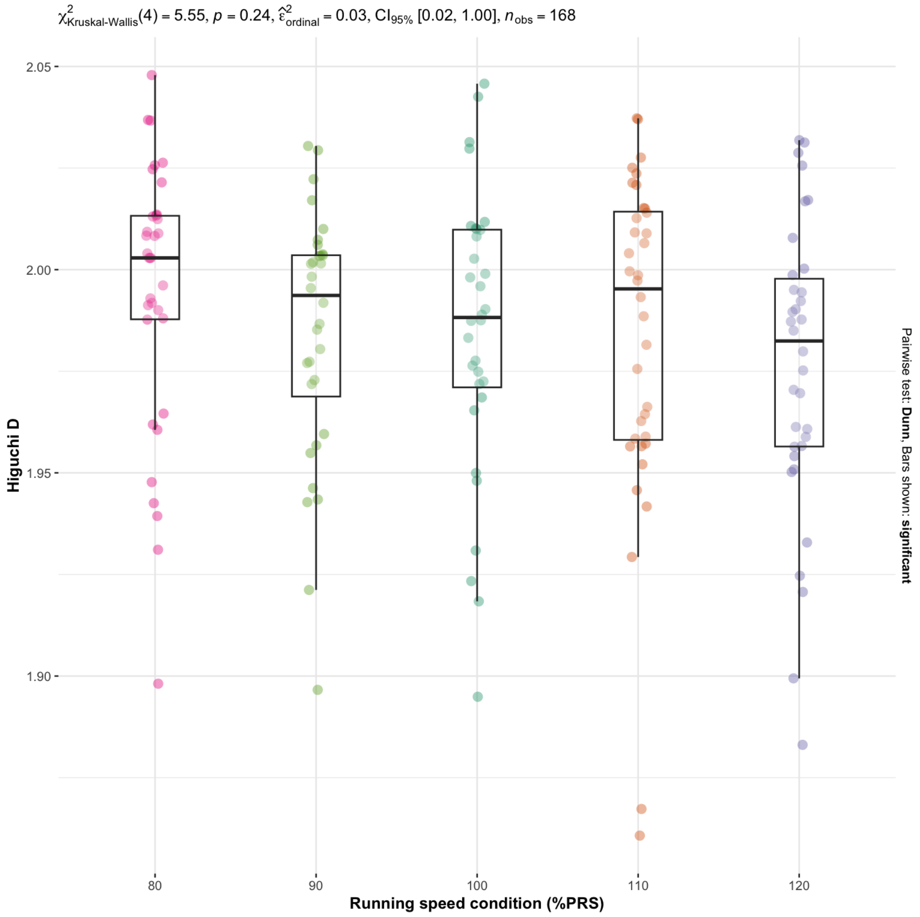

ANOVA and post-hoc tests analysis for DFA-alpha with different running speeds.

Influence of treadmill inclination on stride frequency

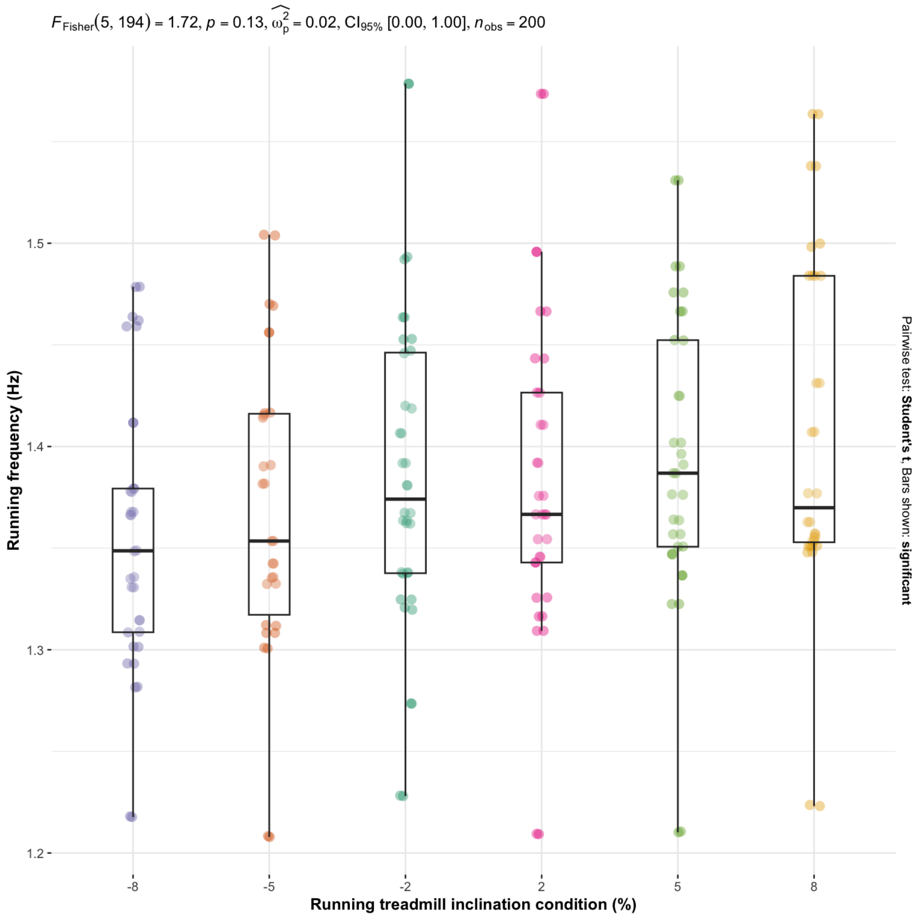

ANOVA and post-hoc tests analysis for running frequency for different treadmill inclinations.

Influence of treadmill inclination on duty factor

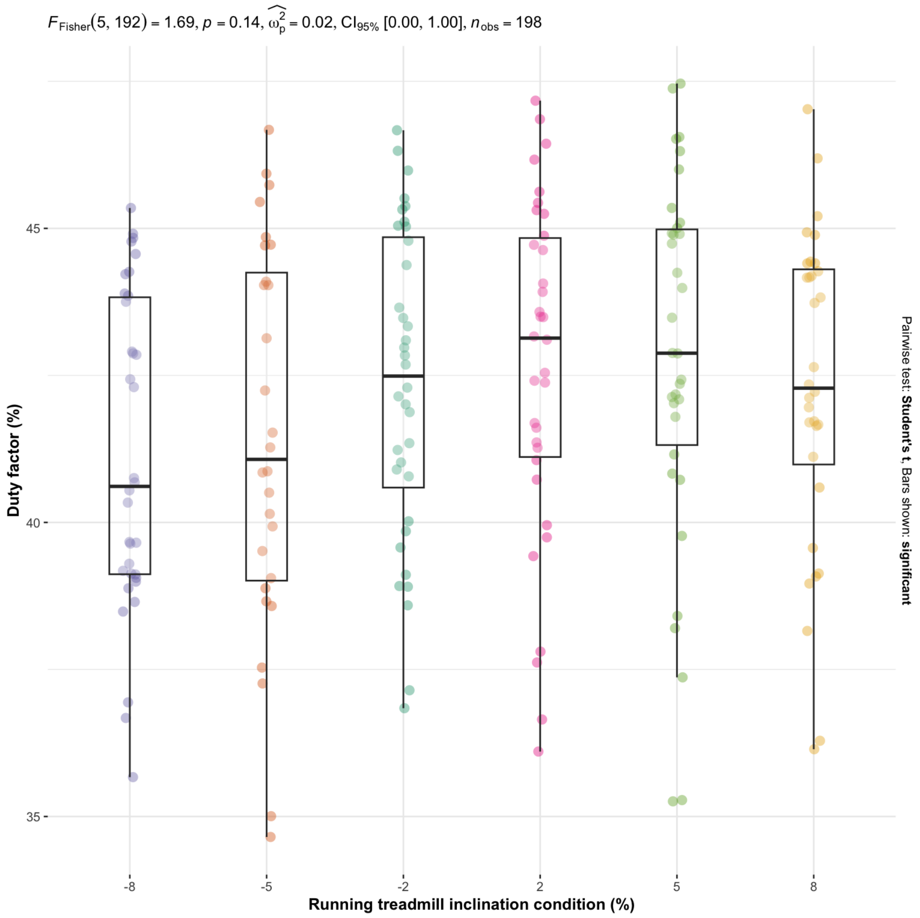

ANOVA and post-hoc tests analysis for duty factor for different treadmill inclinations.

Influence of treadmill inclination on DFA-alpha

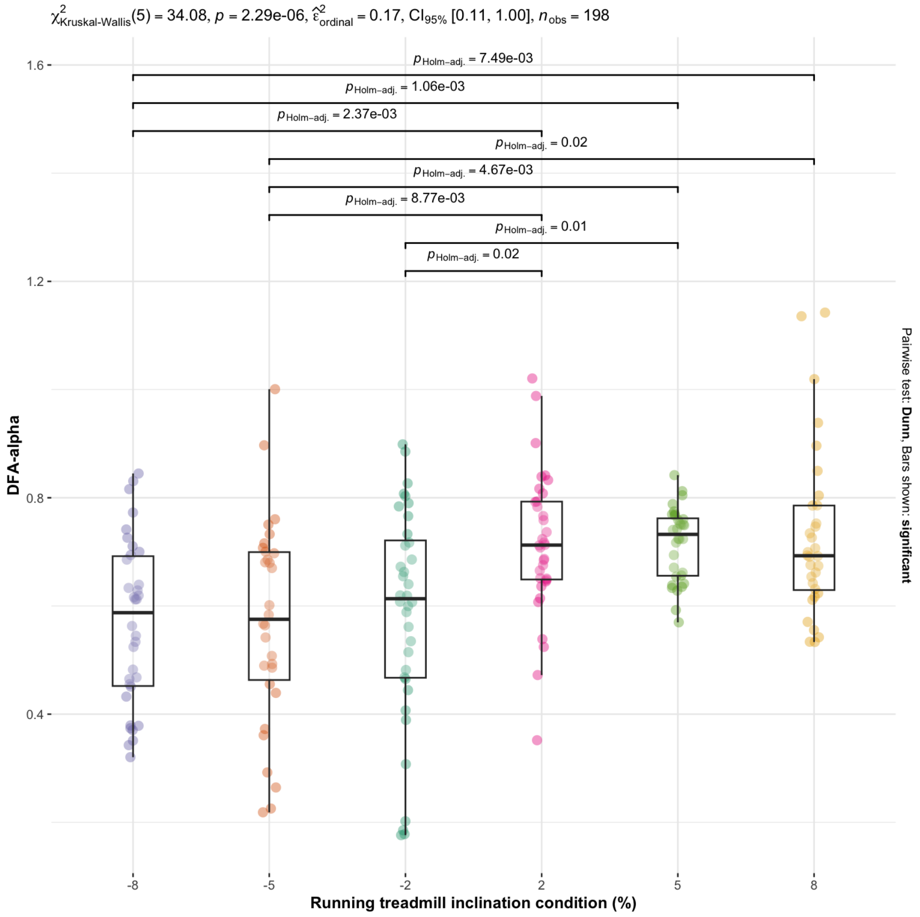

ANOVA and post-hoc tests analysis for DFA-alpha for different treadmill inclinations.

Influence of treadmill inclination on Higuchi's D

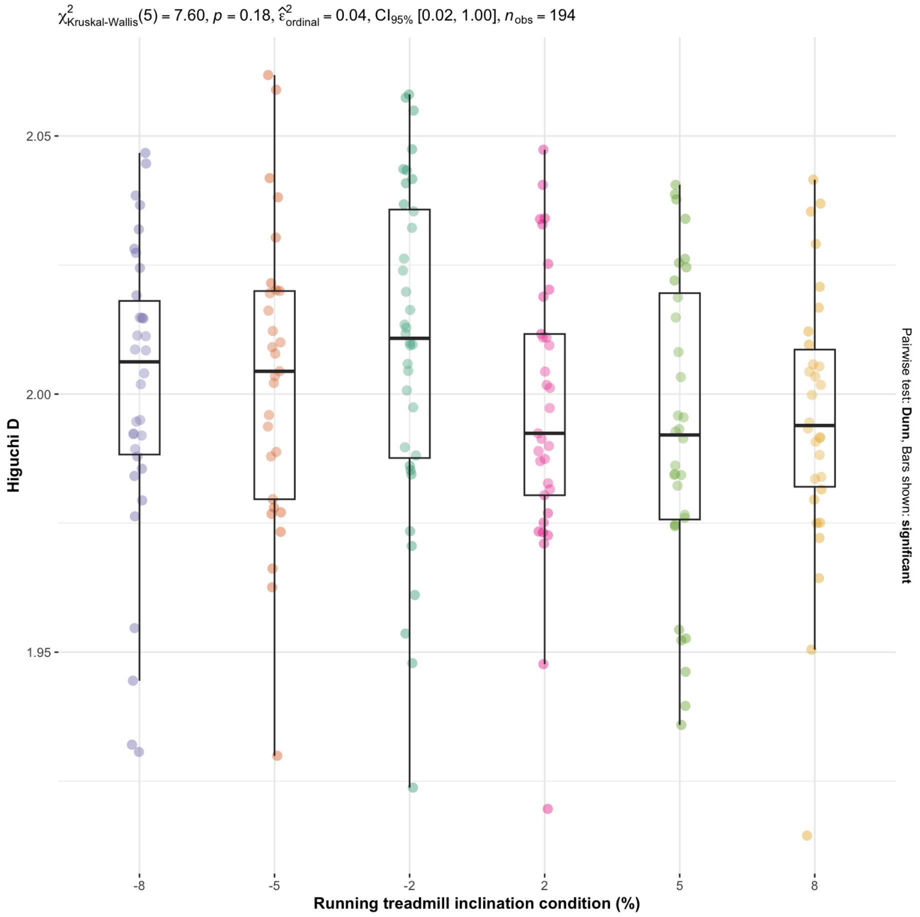

ANOVA and post-hoc tests analysis for Higuchi's D for different treadmill inclinations.

Summary table

Summary statistics											
Entire dataset before splitting (OPTO, MV) or only validation dataset (NN)											
Running cond.	OPTO-p	OPTO-Avg.	OPTO-Sd.	MV-p	MV-Avg.	MV-Sd.	NN-p	NN-Avg.	NN-Sd.	Vars.	Unit
-8	0.147	1.36	0.07	0.010	1.39	0.05	0.032	1.40	0.03	SF	Hz
-5	0.293	1.37	0.08	0.002	1.40	0.05	0.048	1.39	0.04	SF	Hz
-2	0.389	1.39	0.08	0.229	1.40	0.07	0.257	1.39	0.05	SF	Hz
2	0.149	1.38	0.08	0.013	1.41	0.06	0.024	1.39	0.04	SF	Hz
5	0.111	1.39	0.08	0.006	1.41	0.06	0.143	1.39	0.04	SF	Hz
8	0.007	1.41	0.09	0.001	1.42	0.07	0.005	1.39	0.05	SF	Hz
80	0.171	1.36	0.07	0.065	1.39	0.06	0.026	1.39	0.04	SF	Hz
90	0.443	1.37	0.08	0.251	1.39	0.06	0.223	1.39	0.05	SF	Hz
100	0.306	1.39	0.09	0.356	1.41	0.07	0.427	1.38	0.05	SF	Hz
110	0.149	1.40	0.09	0.189	1.44	0.08	0.429	1.40	0.06	SF	Hz
120	0.144	1.44	0.10	0.118	1.46	0.08	0.377	1.41	0.06	SF	Hz
-8	0.041	41.15	2.71	0.257	0.51	0.04	0.043	0.40	0.02	DF	%
-5	0.341	41.42	3.32	0.514	0.50	0.03	0.090	0.41	0.02	DF	%
-2	0.355	42.34	2.66	0.002	0.49	0.02	0.025	0.39	0.03	DF	%
2	0.238	42.64	2.92	0.198	0.50	0.03	0.001	0.40	0.03	DF	%
5	0.060	42.78	3.20	0.046	0.49	0.03	0.024	0.40	0.03	DF	%
8	0.146	42.28	2.71	0.003	0.48	0.02	0.012	0.39	0.03	DF	%
80	0.380	44.78	3.17	0.000	0.50	0.02	0.004	0.40	0.03	DF	%
90	0.047	41.70	3.03	0.043	0.49	0.04	0.026	0.39	0.04	DF	%
100	0.055	40.12	2.83	0.020	0.49	0.05	0.346	0.38	0.03	DF	%
110	0.042	38.10	2.96	0.003	0.49	0.06	0.007	0.38	0.04	DF	%
120	0.036	36.55	2.83	0.001	0.51	0.06	0.061	0.37	0.04	DF	%
-8	0.194	0.57	0.15	0.091	0.55	0.18	0.068	0.80	0.08	DFA	-
-5	0.524	0.57	0.19	0.006	0.59	0.16	0.471	0.75	0.08	DFA	-
-2	0.062	0.58	0.20	0.123	0.56	0.14	0.008	0.84	0.07	DFA	-
2	0.633	0.71	0.13	0.008	0.56	0.20	0.246	0.81	0.08	DFA	-
5	0.216	0.72	0.07	0.004	0.53	0.16	0.313	0.80	0.05	DFA	-
8	0.003	0.73	0.16	0.025	0.51	0.16	0.033	0.80	0.05	DFA	-
80	0.919	0.69	0.10	0.005	0.56	0.19	0.007	0.79	0.06	DFA	-
90	0.146	0.73	0.08	0.179	0.57	0.15	0.168	0.79	0.09	DFA	-
100	0.635	0.73	0.11	0.016	0.52	0.15	0.100	0.79	0.07	DFA	-
110	0.097	0.76	0.13	0.014	0.53	0.18	0.662	0.80	0.08	DFA	-
120	0.000	0.80	0.18	0.088	0.54	0.15	0.320	0.79	0.04	DFA	-
-8	0.062	2.00	0.03	0.340	2.01	0.04	0.003	1.91	0.03	HG	-
-5	0.912	2.00	0.03	0.008	2.00	0.03	0.182	1.92	0.03	HG	-
-2	0.291	2.01	0.03	0.034	2.00	0.04	0.004	1.90	0.03	HG	-
2	0.404	2.00	0.03	0.199	1.99	0.04	0.099	1.91	0.03	HG	-
5	0.237	1.99	0.03	0.241	2.02	0.04	0.132	1.93	0.01	HG	-
8	0.226	2.00	0.03	0.102	2.03	0.03	0.010	1.94	0.02	HG	-

Summary statistics											
Entire dataset before splitting (OPTO, MV) or only validation dataset (NN)											
80	0.018	1.99	0.03	0.069	2.01	0.03	0.162	1.92	0.03	HG	-
90	0.046	1.98	0.03	0.103	2.01	0.03	0.205	1.91	0.03	HG	-
100	0.300	1.98	0.04	0.067	2.01	0.03	0.078	1.92	0.03	HG	-
110	0.002	1.98	0.04	0.001	2.00	0.05	0.127	1.91	0.02	HG	-
120	0.286	1.98	0.04	0.296	2.01	0.03	0.205	1.92	0.03	HG	-

Summary statistics					
Entire dataset before splitting (OPTO, MV) or only validation dataset (NN)					
Running cond.	NN-long-p	NN-long-Avg.	NN-long-Sd.	Vars.	Unit
-8	0.624	0.50	0.07	DFA	-
-5	0.700	0.50	0.06	DFA	-
-2	0.132	0.52	0.09	DFA	-
2	0.702	0.58	0.08	DFA	-
5	0.907	0.59	0.07	DFA	-
8	0.349	0.58	0.04	DFA	-
80	0.255	0.57	0.08	DFA	-
90	0.720	0.60	0.07	DFA	-
100	0.333	0.60	0.08	DFA	-
110	0.924	0.58	0.07	DFA	-
120	0.950	0.58	0.07	DFA	-

Validation dataset (10 random participants)

In this section, details about the comparison between estimation strategies (IMU on the feet and IMU on the thorax + ML algorithm) are given for the testing dataset (n=10) for the stride frequency, the duty factor, the DFA-alpha, and Higuchi's D.

Stride frequency: running condition (speed and slope) and

estimation strategy

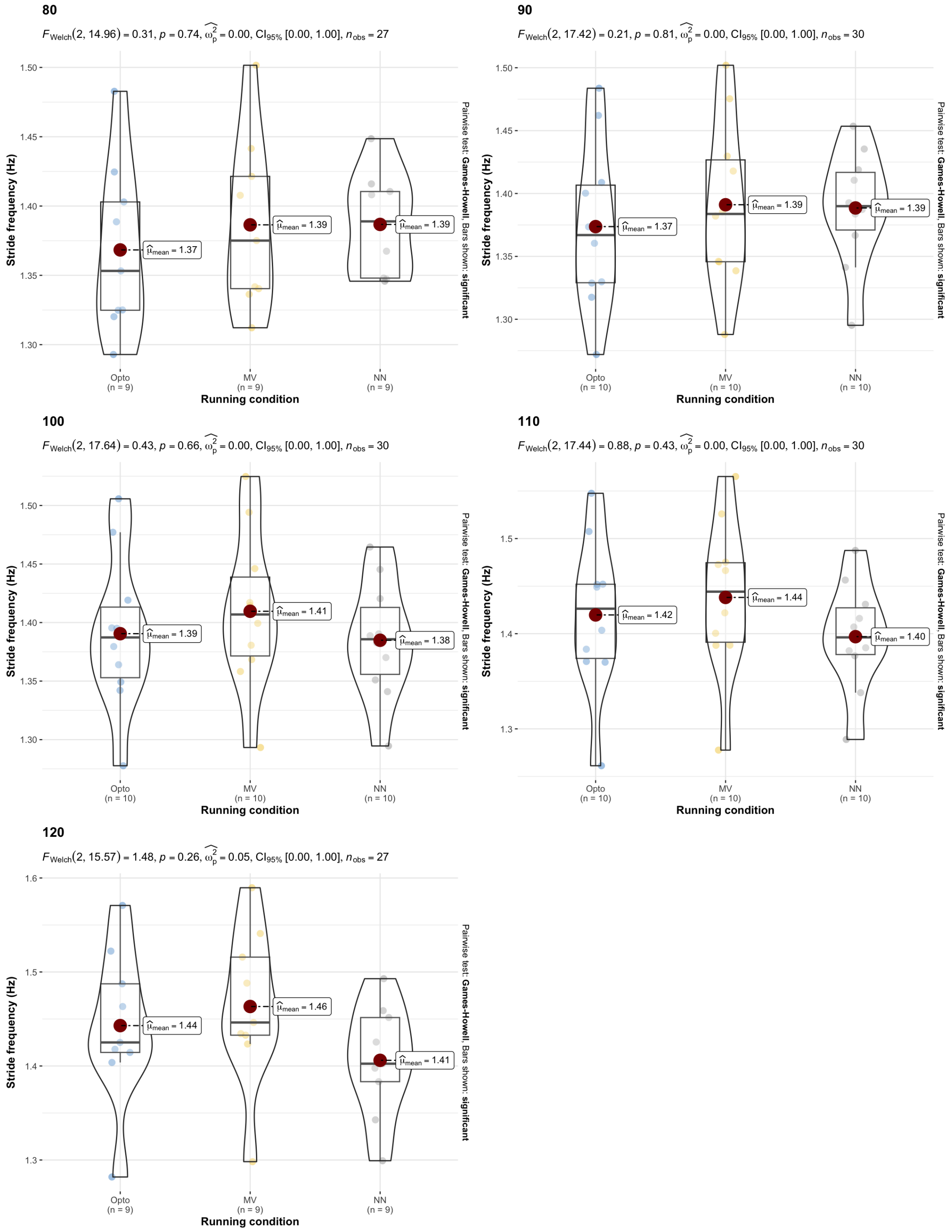

Stride frequency right foot obtained with Optogait (Opto), Movesense sensors placed at the right foot (MV) and Movesense sensor placed at the thorax and used as input for the AI algorithm (NN). Different speeds.

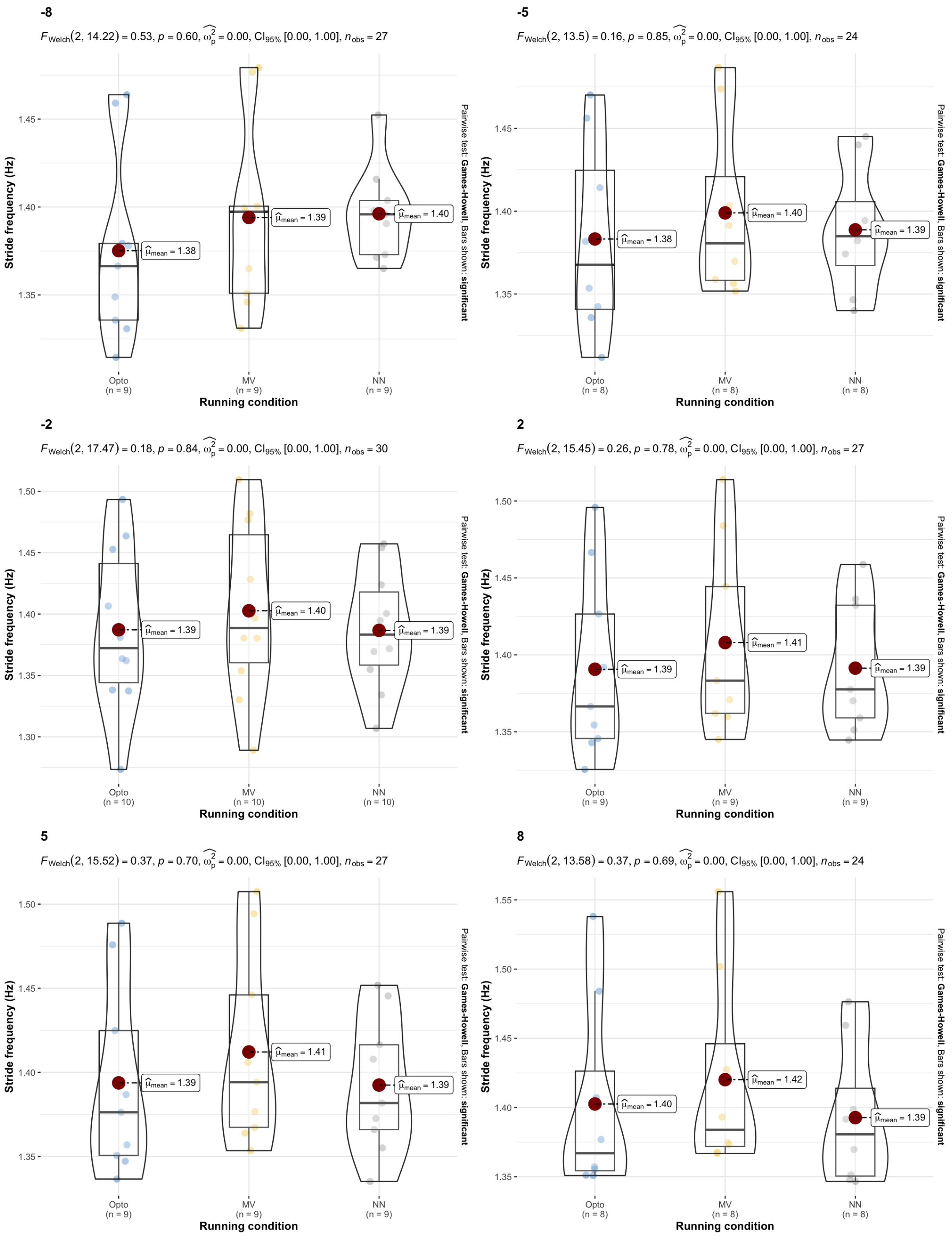

Stride frequency right foot obtained with Optogait (Opto), Movesense sensors placed at the right foot (MV) and Movesense sensor placed at the thorax and used as input for the AI algorithm (NN). Different treadmill inclinations.

Duty factor: running condition (speed and slope) and estimation

strategy

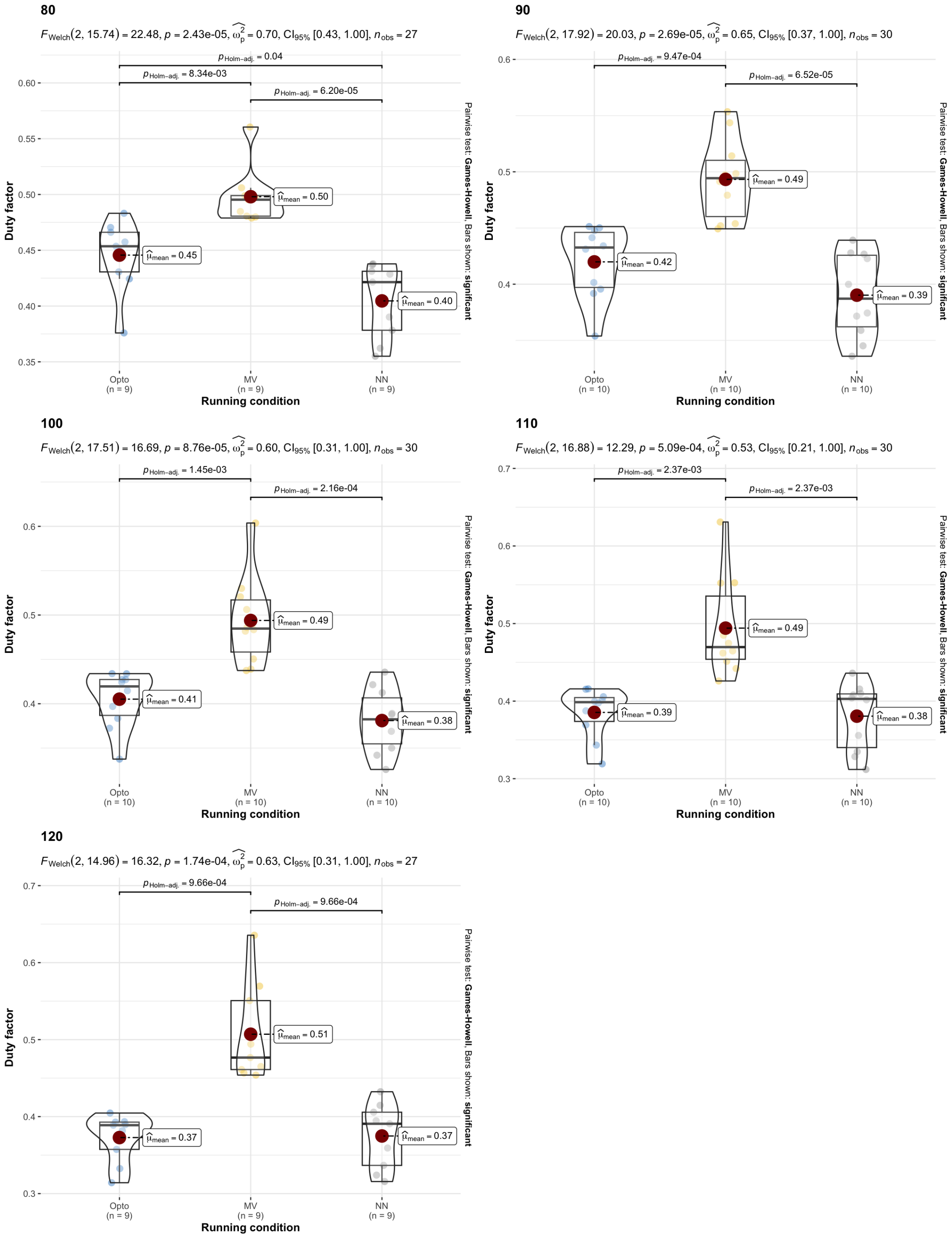

Duty factor right foot obtained with Optogait (Opto), Movesense sensors placed at the right foot (MV) and Movesense sensor placed at the thorax and used as input for the AI algorithm (NN). Different running speeds.

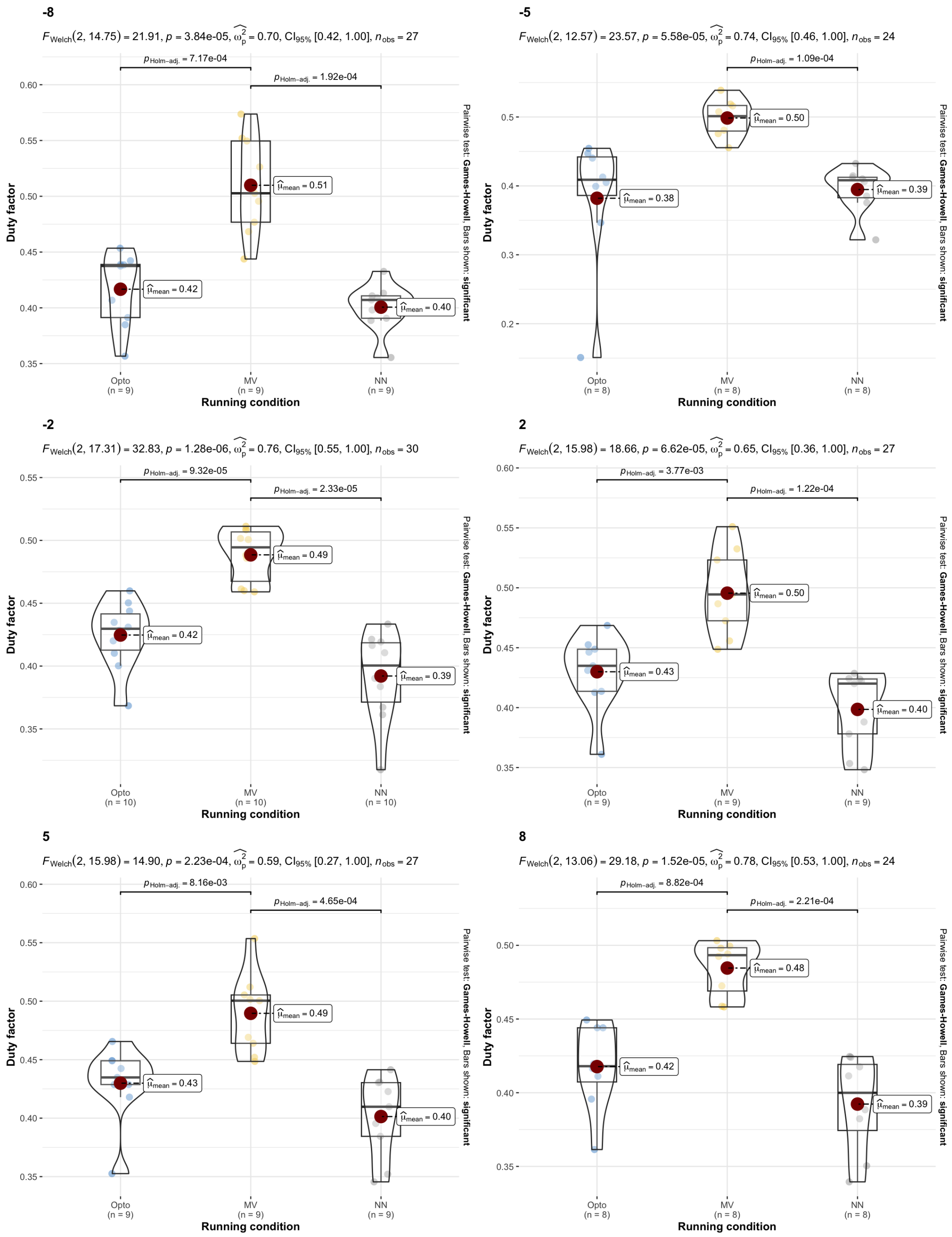

Duty factor right foot obtained with Optogait (Opto), Movesense sensors placed at the right foot (MV) and Movesense sensor placed at the thorax and used as input for the AI algorithm (NN). Different treadmill inclinations.

DFA-alpha: running condition (speed and slope) and estimation strategy

```
## Joining, by = c("cond", "sbj")
```

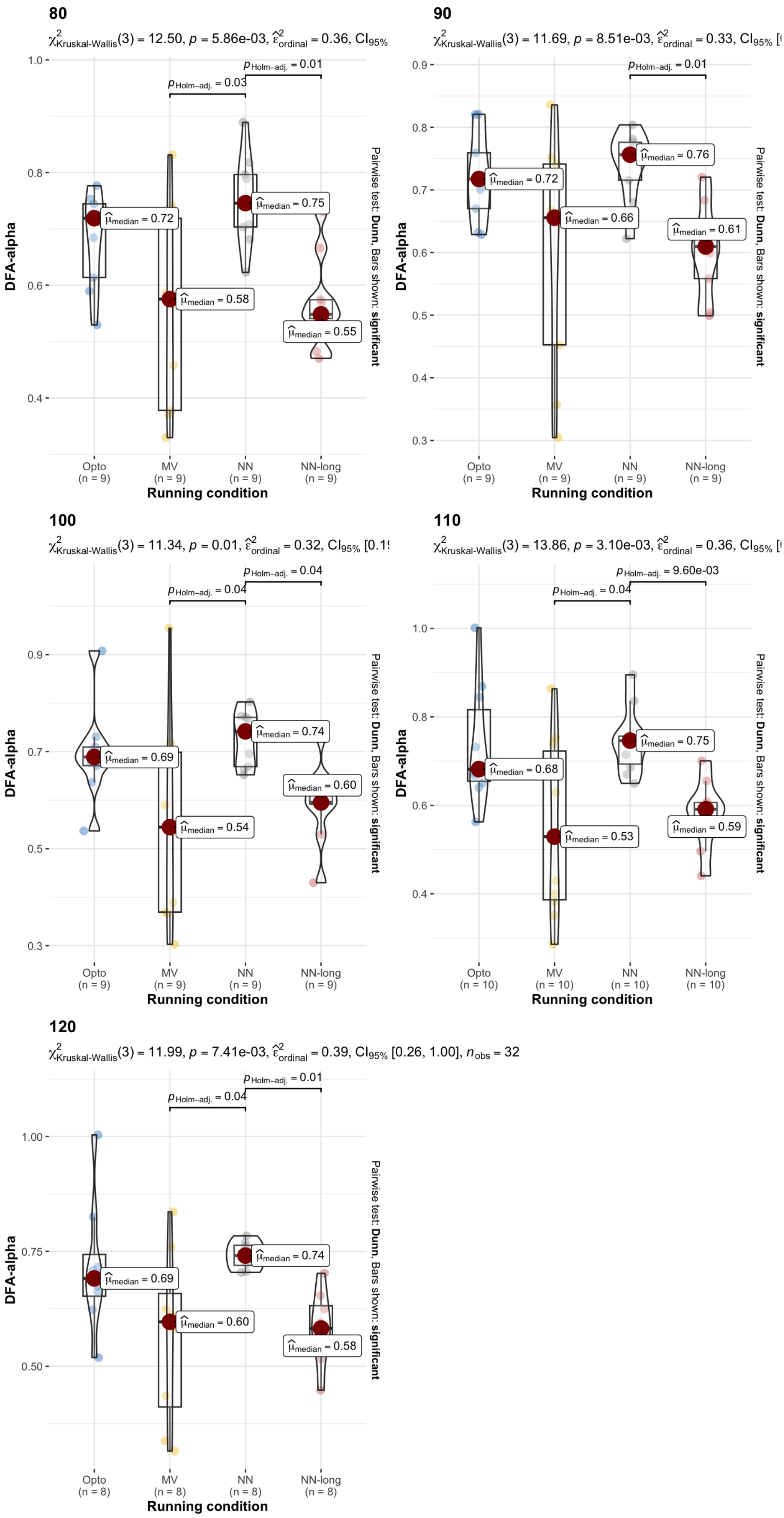

DFA-alpha obtained with Optogait (Opto), Movesense sensors placed at the left foot (MV) and Movesense sensor placed at the thorax and used as input for the AI algorithm (NN). Different running speeds.

```
## Joining, by = c("cond", "sbj")
```

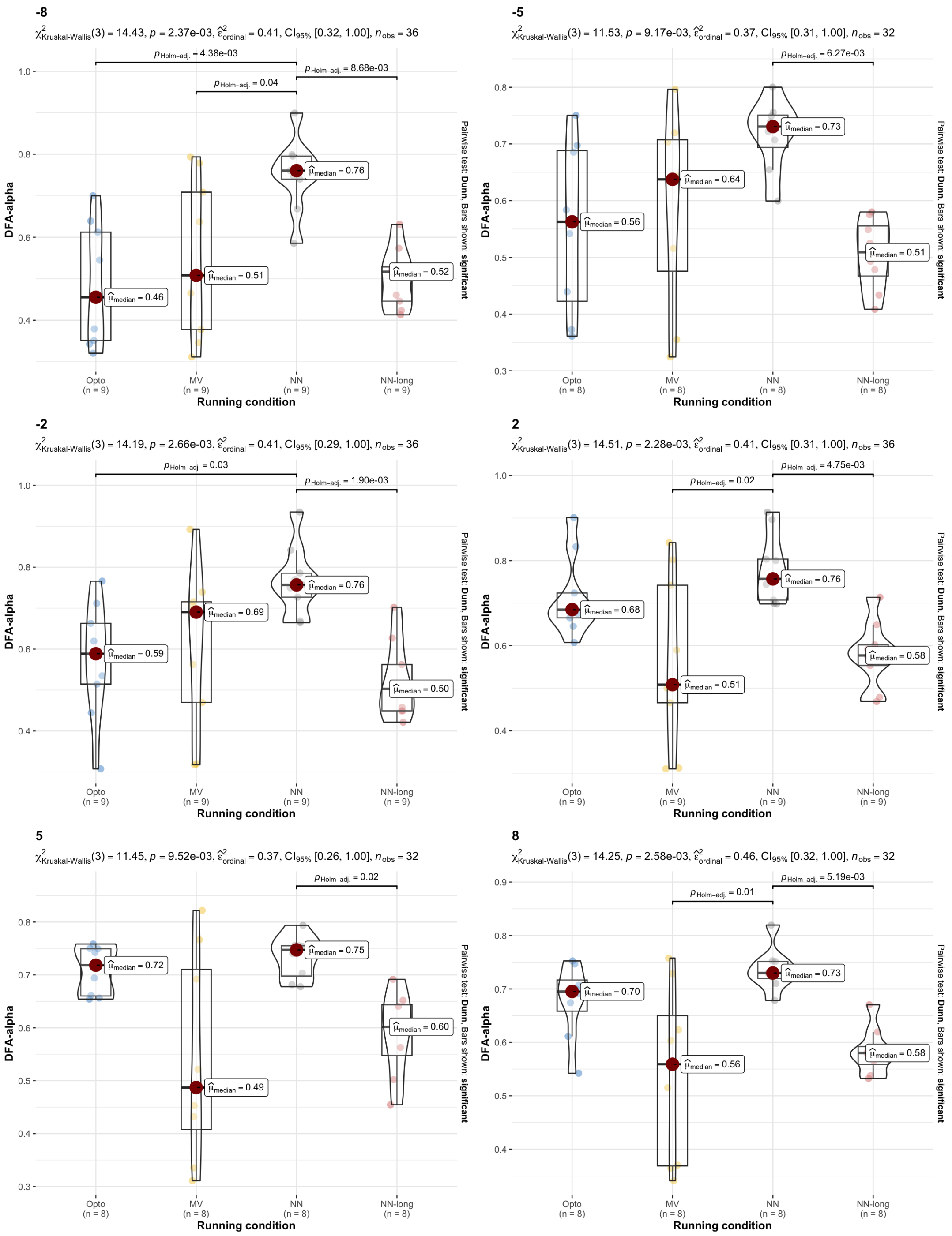

DFA-alpha obtained with Optogait (Opto), Movesense sensors placed at the left foot (MV) and Movesense sensor placed at the thorax and used as input for the AI algorithm (NN). Different treadmill inclinations.

Higuchi’s D: running condition (speed and slope) and estimation

strategy

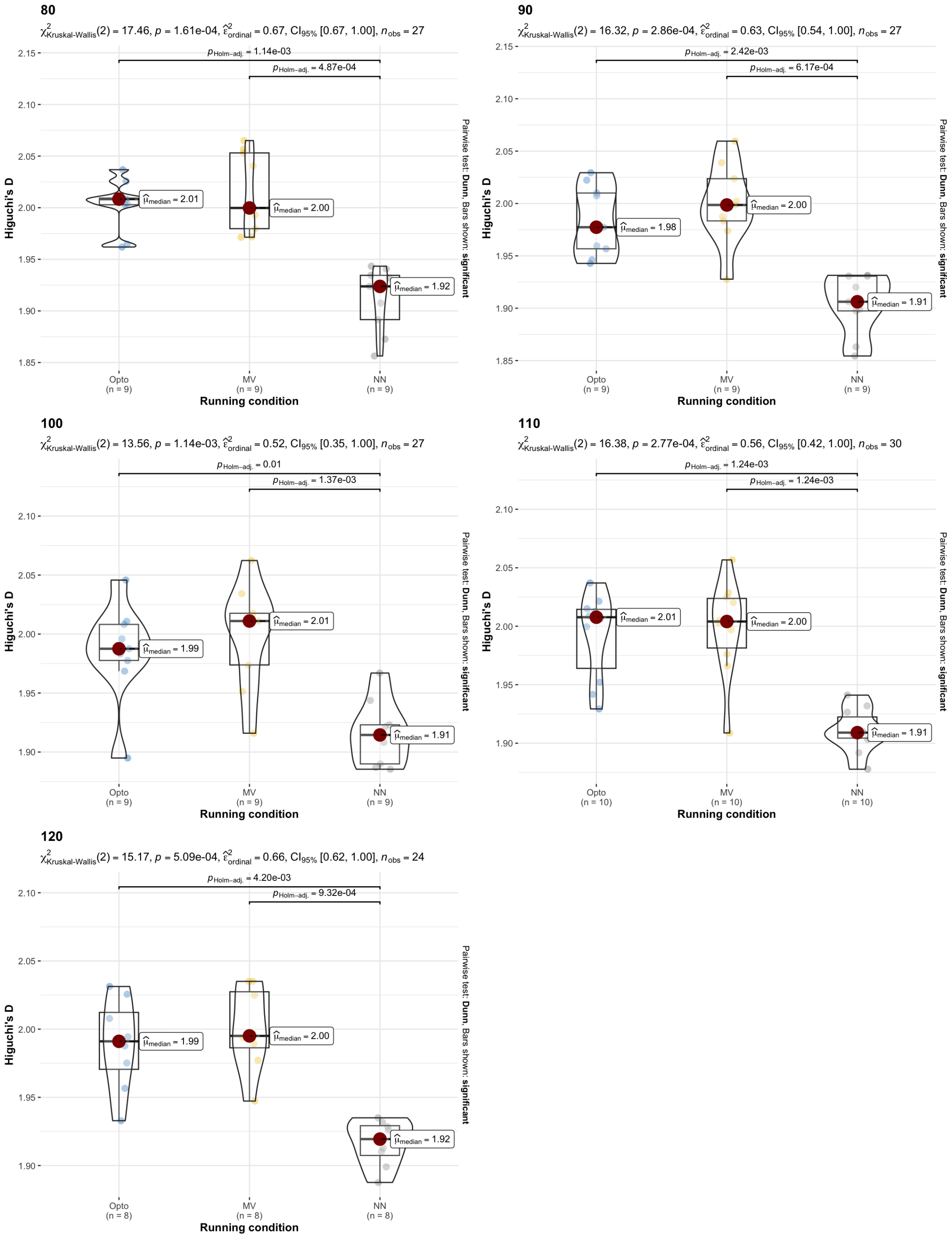

Higuchi s D obtained with Optogait (Opto), Movesense sensors placed at the left foot (MV) and Movesense sensor placed at the thorax and used as input for the AI algorithm (NN). Different running speeds.

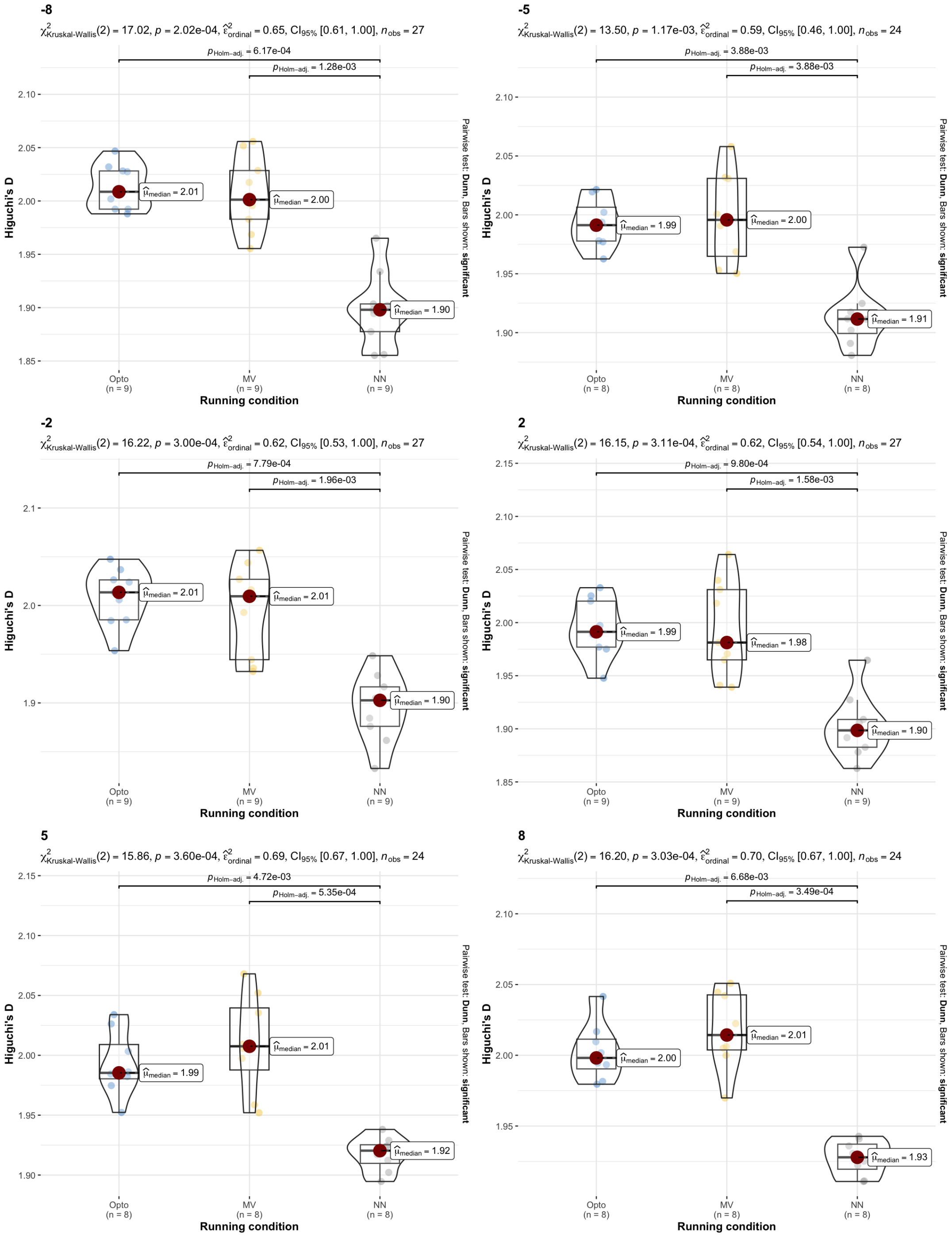

Higuchi s D obtained with Optogait (Opto), Movesense sensors placed at the left foot (MV) and Movesense sensor placed at the thorax and used as input for the AI algorithm (NN). Different treadmill inclinations.

It is common practice to include the average and limits of agreement with horizontal dashed lines in Bland-Altman plots. These limits of agreement have been approximated with ±1.96 times the standard deviation of the difference between the gold standard and the estimated values. Additionally, horizontal lines corresponding to the selected minimum worthwhile difference were included in plots representing the root mean square error.

Specific comparison between estimation strategies IMU on feet versus Optogait

Using the Movesense sensors placed at the foot level to retrieve the stride

frequency and the duty factor

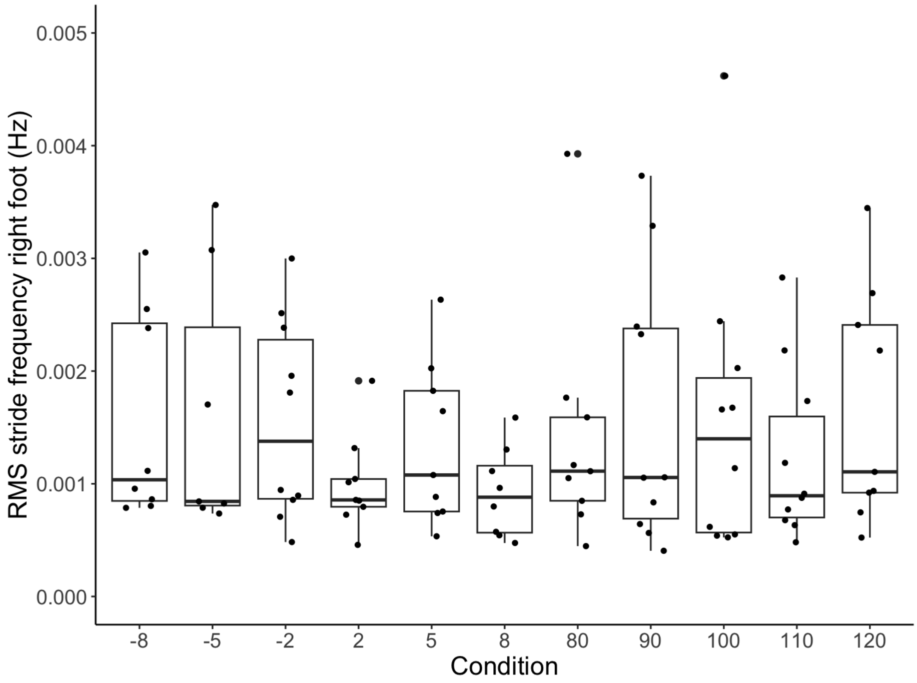

RMS stride frequency right foot. Movesense feet sensors VS Optogait.

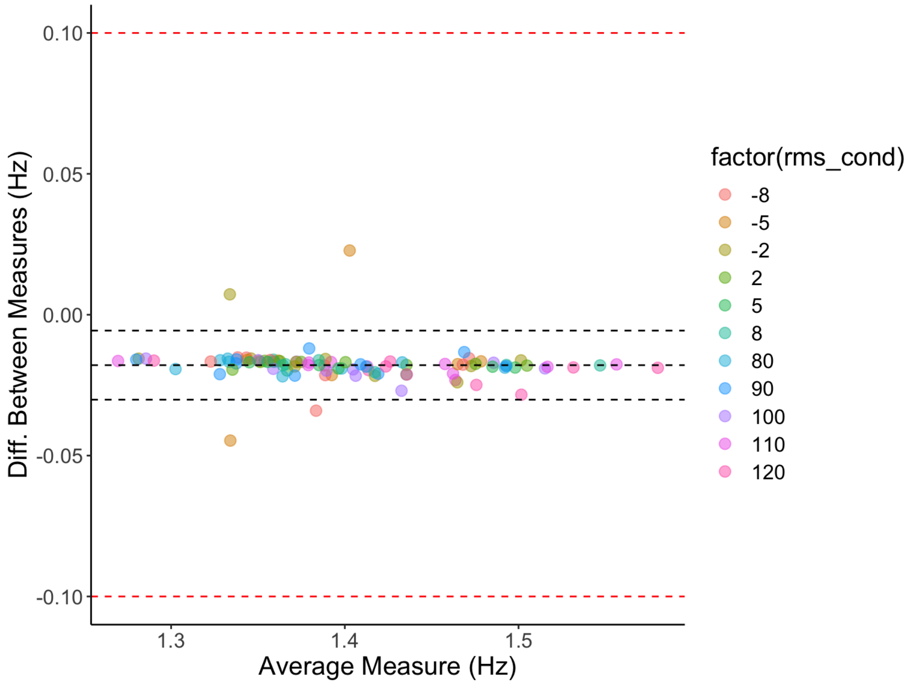

Bland-Altman plot: stride frequency right foot. Movesense feet sensors VS Optogait.

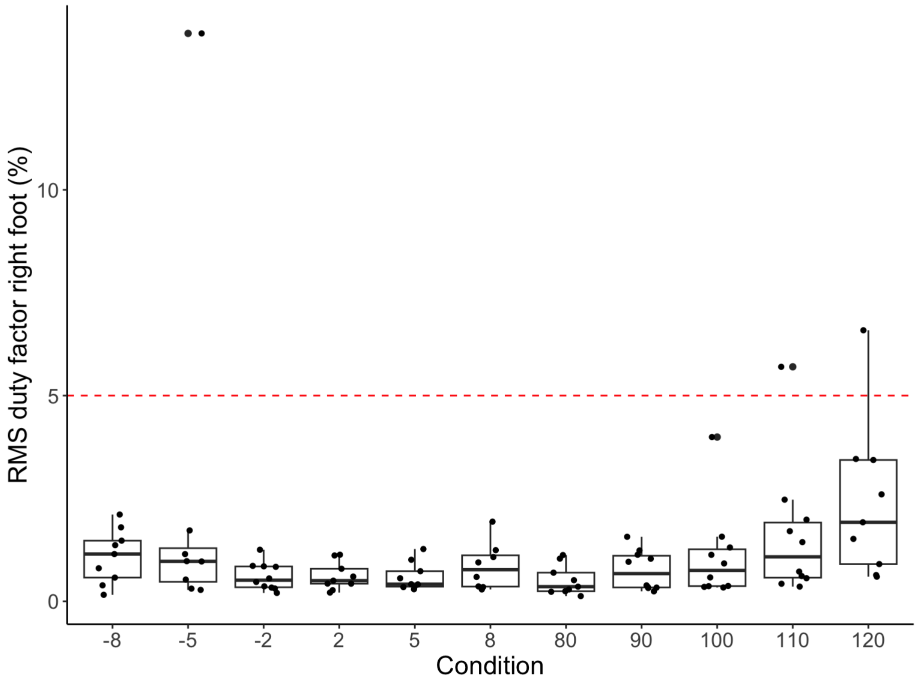

RMS duty factor right foot. Movesense feet sensors VS Optogait.

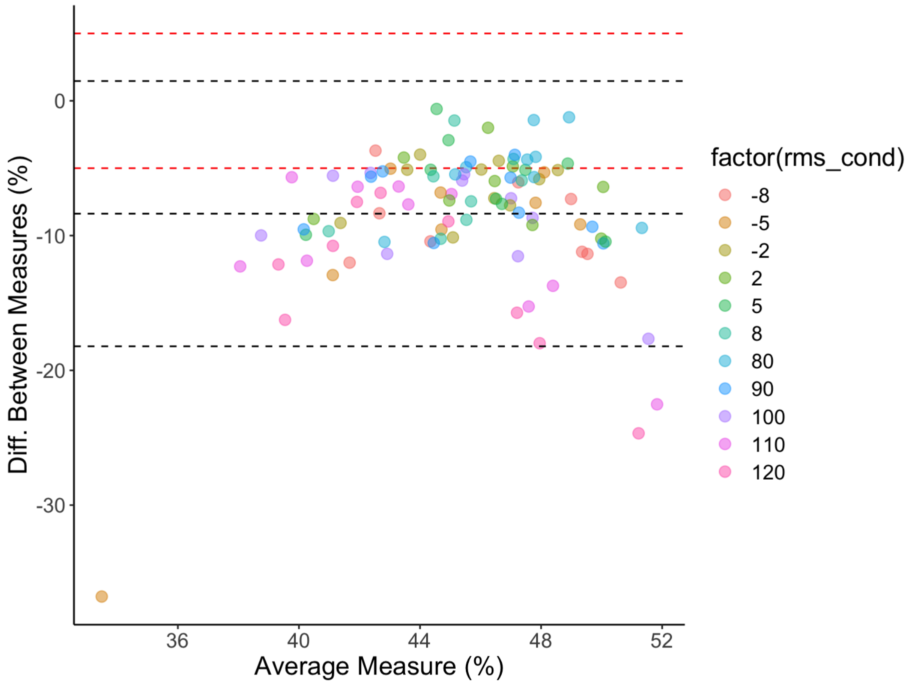

Bland-Altman plot: duty factor right foot. Movesense feet sensors VS Optogait.

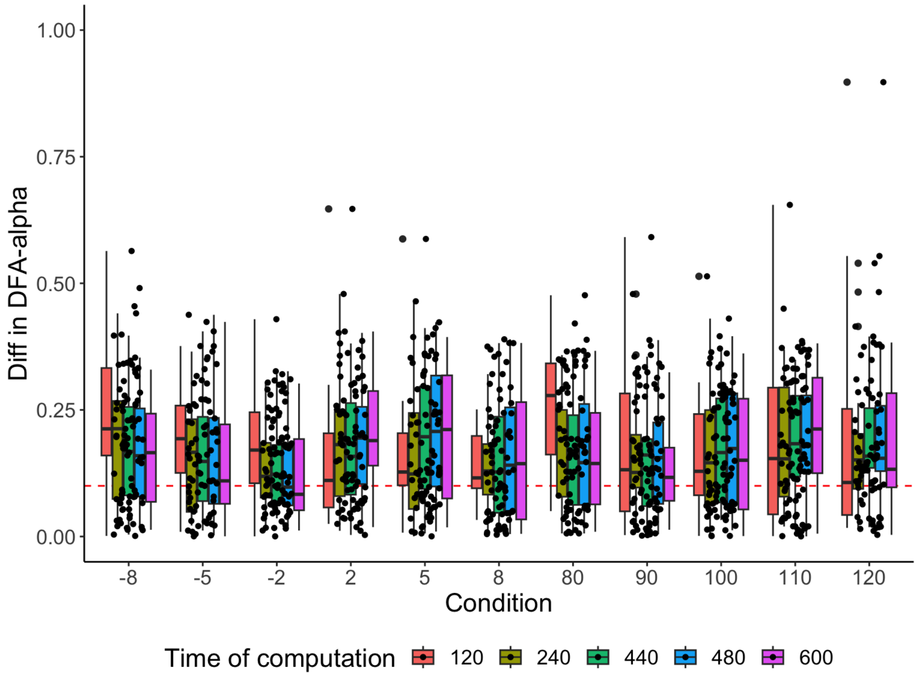

Difference in the calculation of DFA-alpha. Movesense feet sensors VS Optogait.

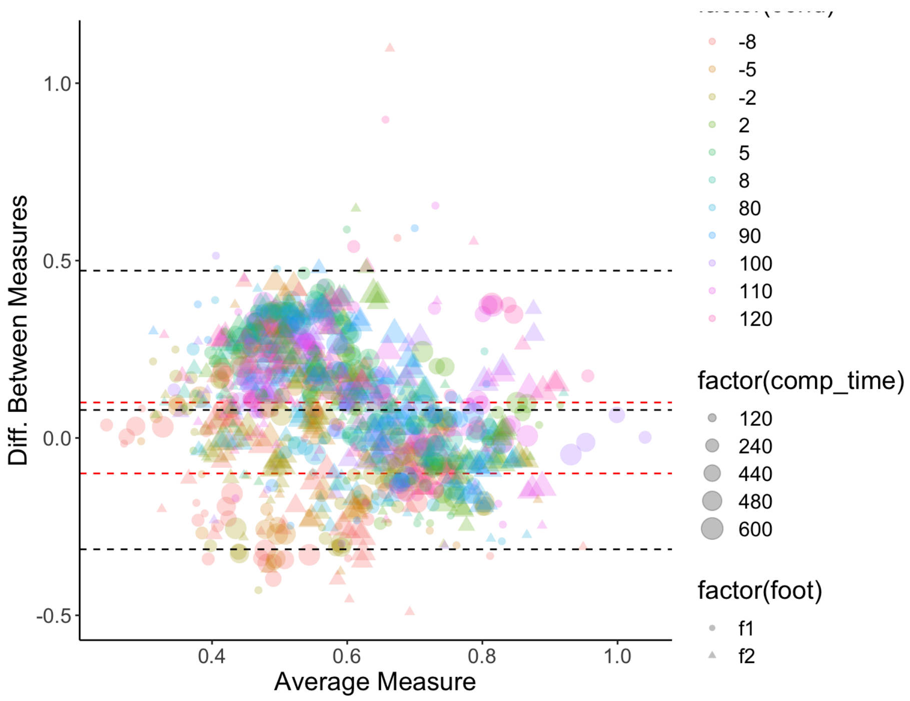

Bland-Altman plot: DFA alpha. Movesense feet sensors VS Optogait.

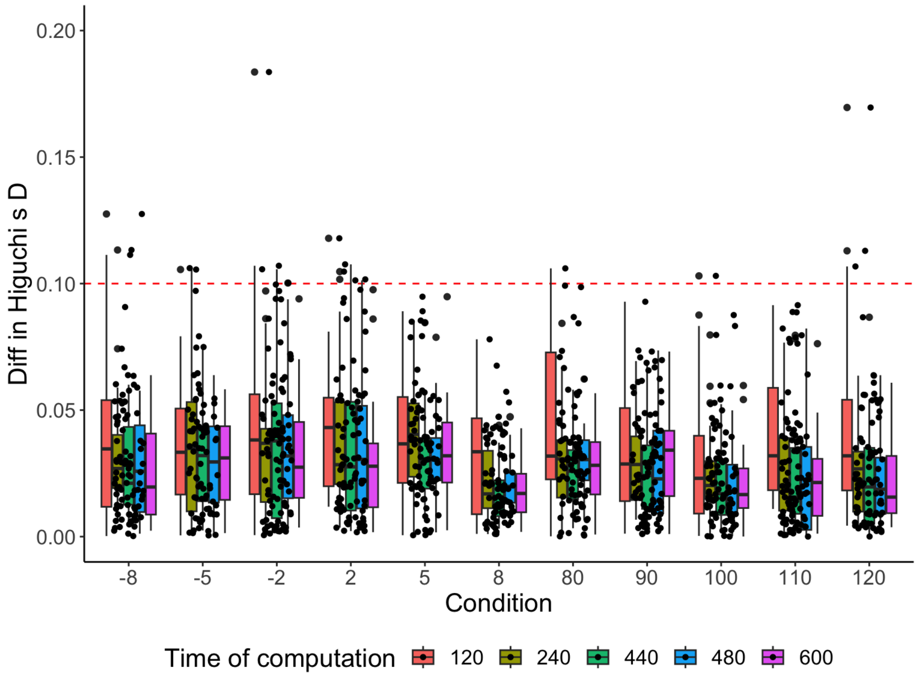

Difference in the calculation of Higuchi s D. Movesense feet sensors VS Optogait.

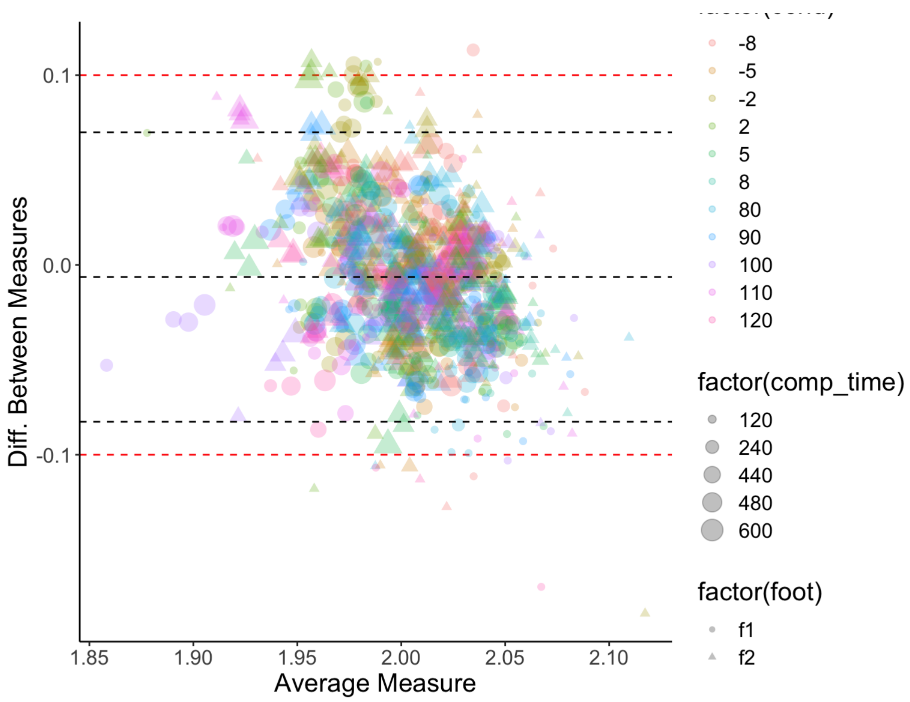

Bland-Altman plot: Higuchi s D. Movesense feet sensors VS Optogait.

Specific comparison between estimation strategies IMU on thorax + ML versus Optogait

Using the neural network model to retrieve the stride frequency and the duty

factor

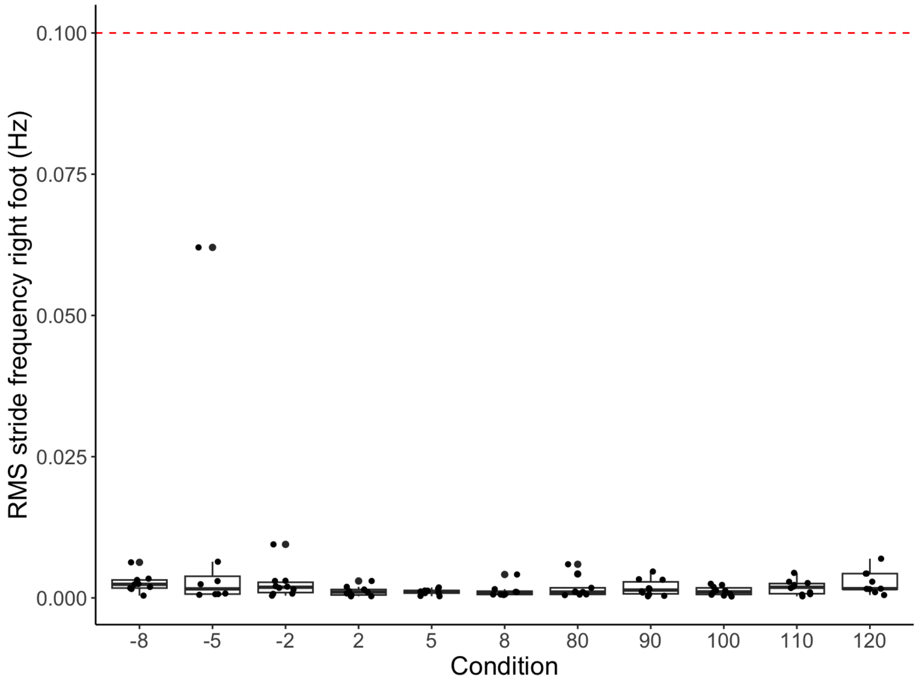

RMS stride frequency right foot. Movesense thorax sensor used as input for neural network VS Optogait.

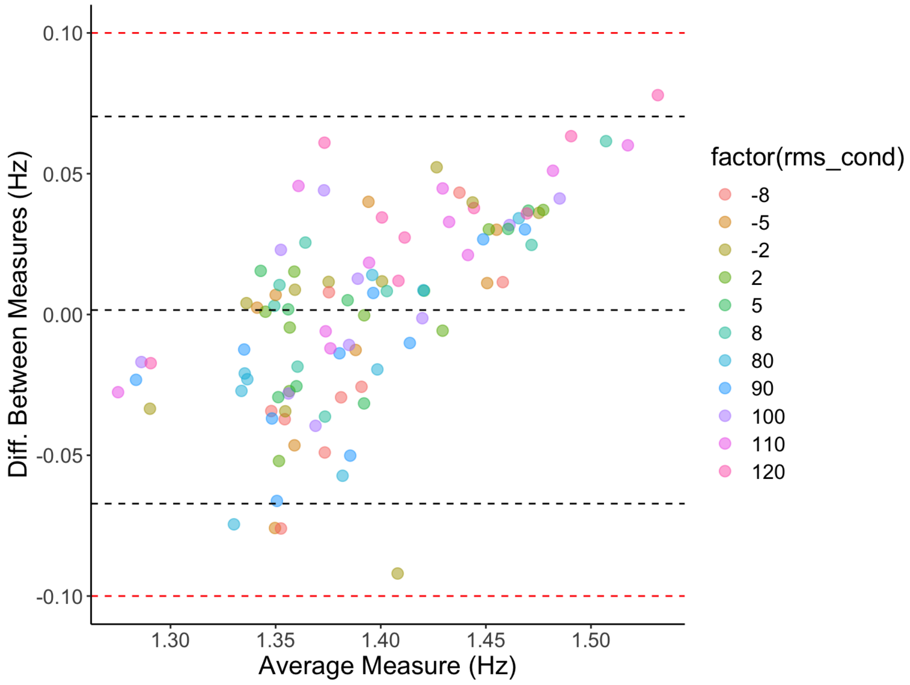

Bland-Altman plot: stride frequency right foot. Movesense thorax sensor used as input for neural network VS Optogait.

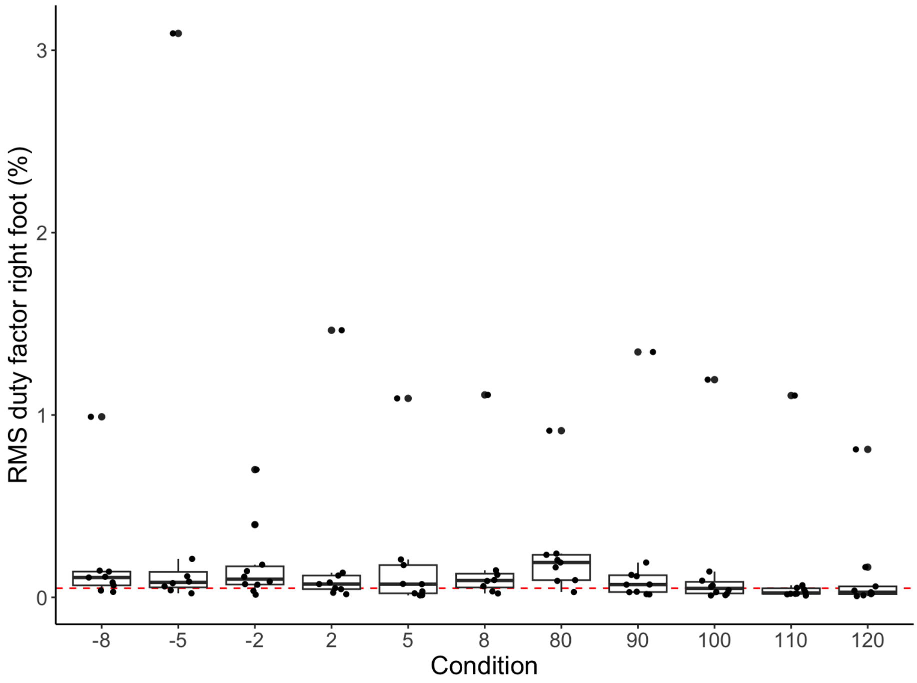

RMS duty factor right foot. Movesense thorax sensor used as input for neural network VS Optogait.

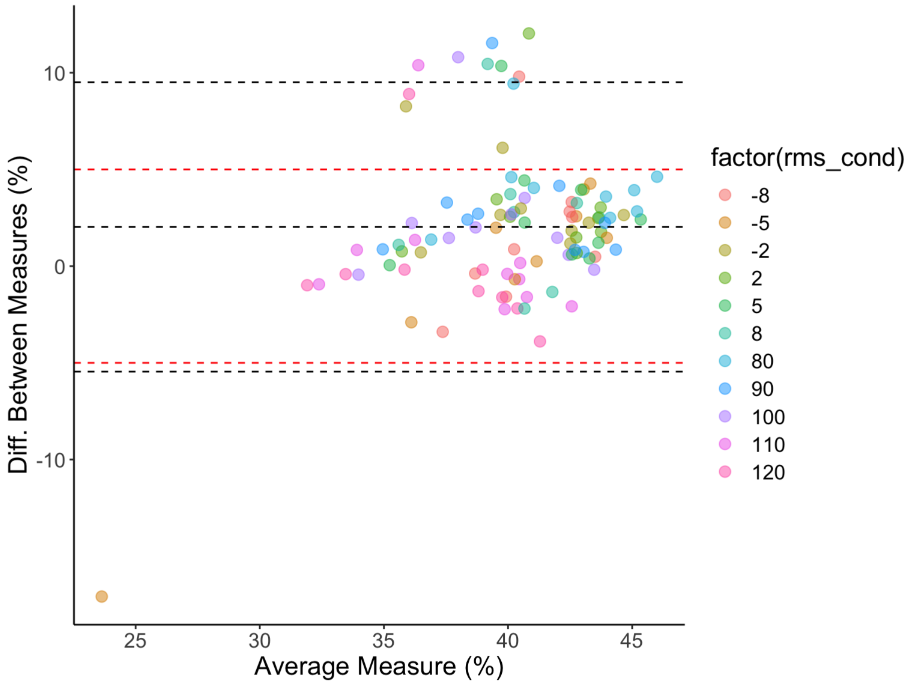

Bland-Altman plot: duty factor right foot. Movesense thorax sensor used as input for neural network VS Optogait.

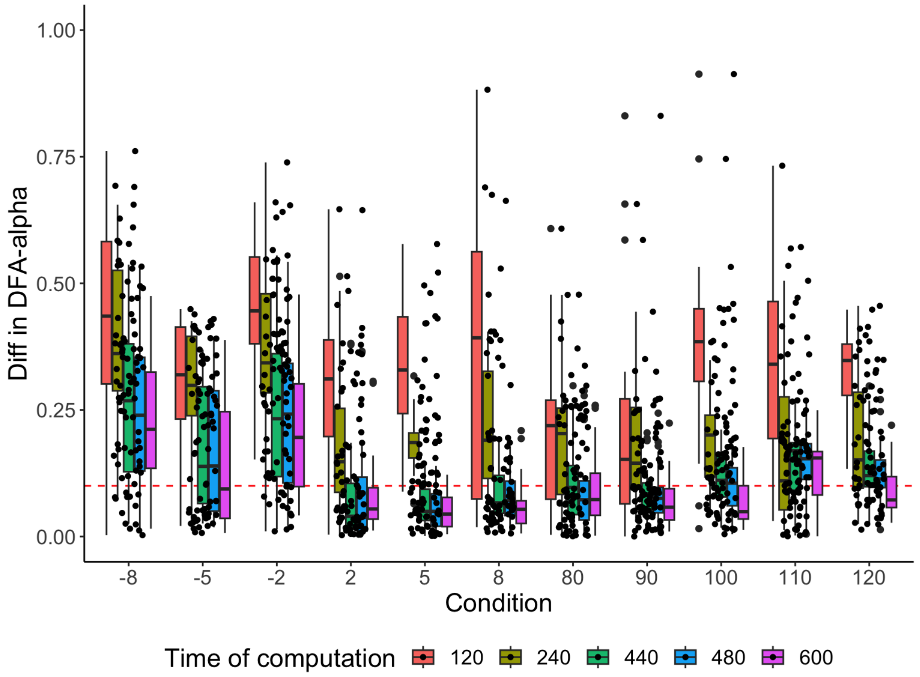

Difference in the calculation of DFA-alpha. Movesense thorax sensor used as input for neural network VS Optogait.

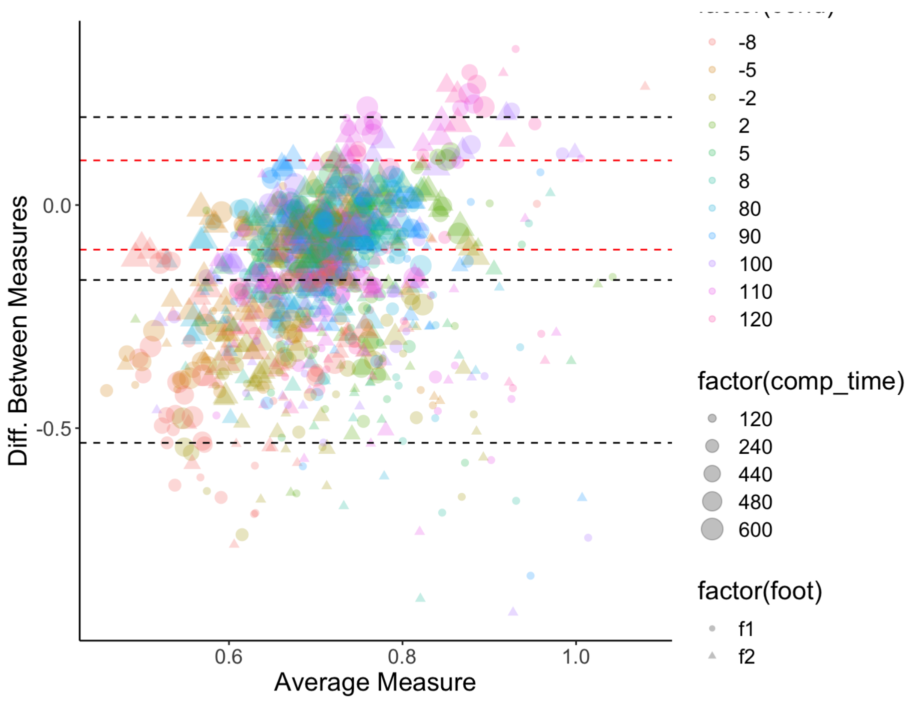

Bland-Altman plot: DFA alpha. Movesense thorax sensor used as input for neural network VS Optogait.

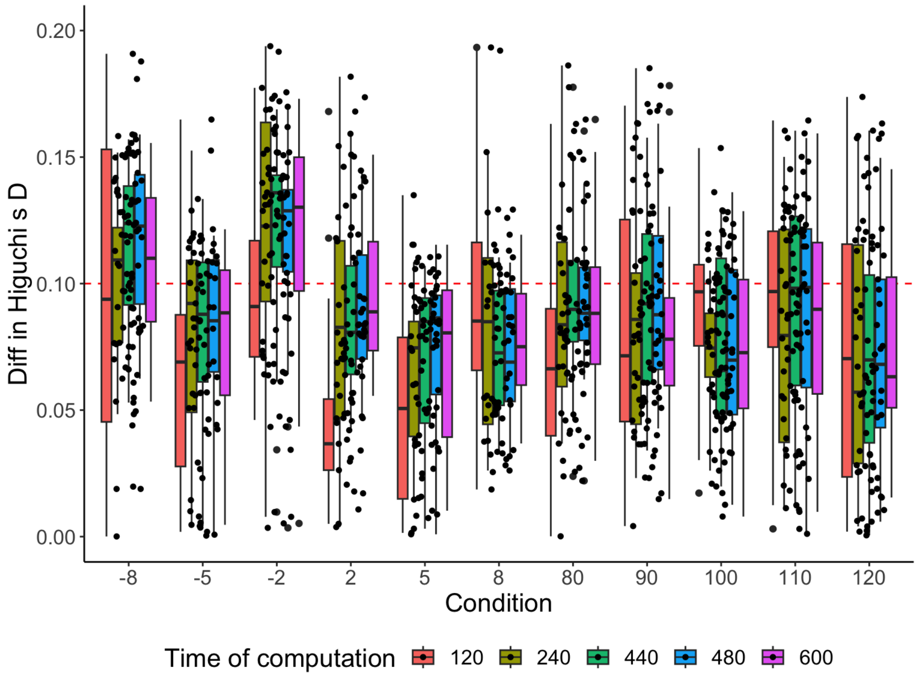

Difference in the calculation of Higuchi s D. Movesense thorax sensor used as input for neural network VS Optogait.

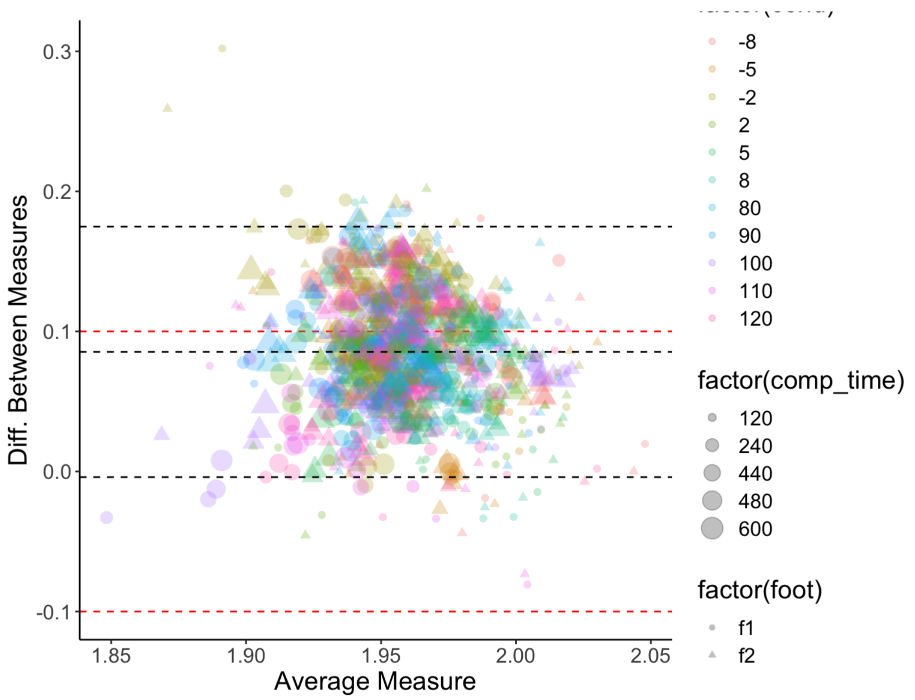

Bland-Altman plot: Higuchi s D. Movesense thorax sensor used as input for neural network VS Optogait.

Summary table

In the following table, differences between estimation strategies are provided with the root mean square error.

```
## Joining, by = "CND"
```

Summary table root mean square errors

Running cond.	SF MV-OPTO (Hz)	DF MV-OPTO (%)	DFA MV-OPTO (-)	HG-D MV-OPTO (-)	SF NN-OPTO (Hz)	DF NN-OPTO (%)	DFA NN-OPTO (-)	HG-D NN-OPTO (-)	DFA NN-LONG-OPTO (-)
-8	0.002	1.092	0.172	0.030	0.003	0.190	0.268	0.115	0.042
-5	0.009	2.467	0.168	0.030	0.010	0.463	0.169	0.079	0.053
-2	0.001	0.577	0.165	0.038	0.002	0.198	0.216	0.114	0.058
2	0.001	0.610	0.199	0.032	0.001	0.224	0.094	0.093	0.042
5	0.001	0.584	0.201	0.036	0.001	0.210	0.043	0.075	0.058

Summary table root mean square errors									
8	0.001	0.850	0.149	0.018	0.001	0.210	0.059	0.075	0.068
80	0.001	0.514	0.199	0.034	0.002	0.240	0.093	0.091	0.046
90	0.001	0.727	0.145	0.031	0.002	0.220	0.065	0.080	0.062
100	0.001	1.041	0.167	0.020	0.001	0.174	0.068	0.070	0.031
110	0.001	1.599	0.209	0.023	0.002	0.138	0.138	0.082	0.036
120	0.002	2.279	0.181	0.023	0.003	0.140	0.100	0.073	0.034

Additional tables

Tables are reported also here to present the results.

In the following table, aggregate results of the RMS in the accuracy of the stride frequency are given. Comparisons have been made between Optical sensor (Optogait, OG) and Movesense sensors at the feet and AI algorithm (NN). Results are given in Hz for both right (R) and left (L) foot. Results have been grouped by indoor running condition. In all conditions, the estimations are deemed accurate.

RMS in SF estimation

Average by running condition (Hz)

Cond.	Movesense-R	NN-R	Movesense-L	NN-L
-8	0.0023	0.0026	0.0011	0.0023
-5	0.0092	0.0096	0.0085	0.0095
-2	0.0016	0.0025	0.0028	0.0024
2	0.0010	0.0012	0.0012	0.0012
5	0.0013	0.0011	0.0054	0.0011
8	0.0009	0.0013	0.0009	0.0013
80	0.0014	0.0019	0.0023	0.0019
90	0.0016	0.0018	0.0024	0.0019
100	0.0016	0.0012	0.0027	0.0013
110	0.0012	0.0018	0.0008	0.0018
120	0.0017	0.0027	0.0010	0.0027

In the following table, aggregate results of the RMS in the accuracy of the duty factor are given. Comparisons have been made between Optical sensor (Optogait, OG) and Movesense sensors at the feet (MV) and AI algorithm (NN). Results are given in %s for both right (R) and left (L) foot. Results have been grouped by indoor running condition. Interestingly, the AI algorithm looks more robust than the feet sensors in the estimation of the duty factor. This is in line with results reported in the literature: estimating contact time with inertial sensors placed at the foot level can only be possible in some running conditions.

RMS in DF estimation

Average by running condition (%)

Cond.	Movesense-R	NN-R	Movesense-L	NN-L
-8	1.092	0.190	1.042	0.152
-5	2.467	0.463	2.507	0.482
-2	0.605	0.181	1.274	0.173
2	0.610	0.224	0.931	0.199
5	0.601	0.189	0.969	0.154
8	0.850	0.210	1.191	0.189
80	0.514	0.240	0.462	0.223
90	0.758	0.201	0.891	0.183

RMS in DF estimation<br>Average by running condition (%)				
100	1.094	0.166	1.428	0.148
110	1.599	0.138	1.723	0.136
120	2.407	0.129	2.134	0.115

In the following table, aggregate results of the RMS in the accuracy of variability indices are given. Comparisons have been made between Optical sensor (Optogait, OG) and Movesense sensors at the feet (MV) and AI algorithm (NN). Results are aggregated for both feet and all conditions, and they are grouped by computational time.

RMS in variability estimation

Average by computational time (sec) between optical sensor (OG), foot sensors Movesense (MV) and ML algorithm (NN)

Comp. Time	Higuchi D MV/OG	DFA-alpha MV/OG	Higuchi D NN/OG	DFA-alpha NN/OG
120	0.039	0.191	0.081	0.336
240	0.032	0.168	0.086	0.232
440	0.027	0.172	0.089	0.140
480	0.028	0.173	0.089	0.131
600	0.027	0.169	0.088	0.114

Appendix

Additional reading and resources

The goal of this report was to provide an overview of the project and of the main results obtained. Many details about the AI algorithm architecture and the data acquisition were not provided here. Additional readings and resources can be found at the following links:

- Repository (https://bitbucket.org/andrea_zignoli/prissiv/src/np/): code and web app documentation
- Web-app (https://andreazignoli.shinyapps.io/view_data/): inference web app
- Wandb (https://wandb.ai/andrea_zignoli/prissiv?workspace=user-andrea_zignoli): training and testing the AI algorithm
- Miro board (https://miro.com/app/board/o9J_lyfD5qs=/?invite_link_id=147204803581): project structure
- Contacts: andrea.zignoli@unitn.it (mailto:andrea.zignoli@unitn.it) & laurent.mourot@univ-fcomte.fr (mailto:laurent.mourot@univ-fcomte.fr)
