# Supplementary material for: Indoor running temporal variability for different running speeds, treadmill inclinations, and three different estimation strategies
Source: PLoS One. 2023 Jul 20;18(7):e0287978. doi: 10.1371/journal.pone.0287978 (PMC10358961; doi:10.1371/journal.pone.0287978)
Supplement: Attachment — Submitted filename: PONE-D-23-07146_review - first round.pdf [file pone.0287978.s002.pdf]

Review – Plos One- PONE-D-23-07146

Title: Indoor running spatio-temporal variability: normative values for different running speeds, inclinations, and three different estimation strategies

Authors: Andrea Zignoli, PhD, M. Eng.; Antoine Godin; Laurent Mourot

Reviewer's comments

The paper analyses the results obtained in terms of stride frequency, duty factor, and detrended fluctuation analysis and Higuchi's D index of the stride frequency obtained during indoor running at different speeds and treadmill inclinations. In general, the paper has a high-quality English language, but the overall structure must be improved, the results must be more elaborated, and the discussion must be clearer.
The authors declared to accomplish two aims: *i*) provide normative values, *ii*) compare three different strategies. However, the first aim is only discussed in the additional materials.
In the Results the authors should revise the reported values and sentences.
I would recommend accepting the manuscript only if the following points will be addressed.

Title

1) I would not mention 'spatio-temporal variability' since the only two estimated running parameters are the stride frequency and the duty factor, which are both only temporal.
2) Are 20 subjects sufficient to provide normative values of the estimated quantities? The authors should clarify that.
3) According to the previous points, I suggest changing the title in 'Indoor running temporal variability for different running speeds, treadmill inclinations, and three different estimation strategies'.

Abstract

1) The authors mention the accuracy of the machine learning algorithm using the IMU on the thorax in the estimation of the duty factor, but the paper is focused on the estimation of the variability indices. I suggest to briefly mention the main results obtained in the estimation of the detrended fluctuation analysis and Higuchi's D index, specifying which of them is more accurately estimated with the IMU on the thorax or on the feet.

Introduction

1) The introduction of the variability indices is discussed nicely. Although, the introduction of the estimated temporal parameters is quite missing. The information required to estimate the stride frequency and the duty factor are the running events (initial and final contact instants). I do recommend citing some studies assessing running events, stride frequency, and duty factor using different technologies.
2) The authors mentioned some technologies confined in the laboratory setting in the following clause: ''Running variability is indeed best assessed in laboratory conditions, where optoelectronic devices, force platforms, motion capture systems, or pressure insoles are available''. Although, pressure insoles are wearable and portable. A study estimating the running cycle (stride) durations in-the-field with pressure insoles is discussed in a recent paper: https://doi.org/10.1111/sms.14341. Thus, I do recommend to explicitly mention that running temporal parameters can be assessed in laboratory conditions using motion capture systems, force platforms, or instrumented treadmills, while, in outdoor conditions, IMUs are typically the best alternative, since they can provide the kinematic of the body segments where they are attached, and pressure insoles can be a portable reference for temporal parameters [1].

3) The reference values are reported only in the additional materials and are assessed only on 20 runners. I suggest mentioning a single general aim of the study: the estimation of stride frequency, duty factor, and associated variability using three different strategies.

Methods

1) *Normative values (Optogait):* Were the DFA-α and Higuchi's D values calculated for both stride frequency and duty factor? It seems that they were calculated only on a single parameter (stride frequency). The authors should clarify that.
2) *Other estimation strategies (IMU on the feet):* The description of the method with IMU on feet should be improved. The authors mentioned a reference study, but they modified it after a trial and error approach. Thus, it is preferable to explicitly describe how the initial and final contacts were found, since they are crucial for the calculation of stride frequency and duty factor.
   A reference to Figure 1 can be added.
3) *Figure 1*: Which inertial signals are shown? Please show the acceleration norm (which is used to estimate the running events from the feet) and highlight not only the initial contact instants but also the final contact instants in the figure.

Results

1) Please add the explicit information of the result of the normality test for each tested distribution.
2) Reference and estimated quantities are only reported in the Additional Materials. In the Results section only the statistical analysis is described. Please add a table or a brief description of the estimates of stride frequency, duty factor, variability indices, and their errors according to the different running conditions. It will increase the readability of the paper and the understanding of the statistical analysis.
3) *Normative values:* The following sentence is reported: ''ANOVA conducted on running frequency with different treadmill inclinations returned **p=0.1322**, so the running frequencies **are dependent** from treadmill inclination''. Please revise the results of the statistical analysis, in particular the above one.
4) *Other estimation strategies:* The following sentences are reported: ''The p-value for the interaction between running condition and estimation strategy is 0.9052, which indicates that the stride frequency value does not depend on the estimation strategy'' and ''The p-value for the interaction between running condition and estimation strategy is 0.9998, which indicates that the stride frequency value does not depend on the estimation strategy''. Please revise and rephrase those sentences. Can ''the p-value for the interaction between running condition and estimation strategy is XXX, which indicates that there is no interaction between running speed/treadmill inclination and estimation strategy'' be more suitable?
5) Sometimes the authors refer to the significant differences between the estimations with the IMU on the feet/thorax and the other two estimation strategies. These kind of information are preferable to be inserted in the Discussion, instead of the Results. Furthermore, the mentioned quantities and errors are only reported in the Additional Materials. I suggest to explicitly mention the values of the obtained errors to help the reader to understand if some differences are significant but not relevant or vice versa.
6) Please revise the following sentence ''Two-way ANOVA conducted on **Higuchi's D** with different treadmill inclinations returned p=0.364 for the treadmill inclination factor and p<0.0001 for the estimation strategy, so the hypothesis that **all duty factor values** are equal for different estimation strategies is rejected. This is due to the significant differences between the estimations with the **IMU on the feet** and the two other estimations strategies''. It seems to be not in accordance with the results reported in the Additional Materials (which highlighted that the IMU on the thorax worsened the results) and to include some typing errors.

Discussion

1) Is the goal to present normative values for indoor running kinematics and variability achieved? I suggest to remove this first aim, as explained before.
2) The comparison with the results in the literature of interest in terms of DFA-α can be improved. If you declare to provide normative values, it might be crucial the comparison with other refence values in the literature. What quantitative normative values are reported in the literature? Which are the studies reporting normative values similar to the ones obtained by the authors? Please report the quantitative differences between the values obtained by the authors and other studies in the literature. It can helpful to know the ranges to understand how much they overlap.
3) The comparison with the results in the literature of interest in terms of stride frequency and duty factor estimated from IMUs on feet or thorax is missing. Are the obtained errors comparable with similar studies? In addition, see the consideration raised above for DFA- α.
4) The comparison with the results in the literature of interest in terms of Higuchi's D index is missing. Are there any studies estimating this variability index in running? Are the results similar? In addition, see the consideration raised above for DFA- α.
5) Are there any other studies investigating variability varying speed and/or surface inclination? Please cite them. The findings of statistical analysis is in accordance with the literature in terms of the observed significant (or not) dependances of the variability indices on running conditions?
6) The following sentence is reported: ''In the estimation of the duty factor, the estimation strategy with the IMU on the feet was found to be not accurate across all the different running speeds and treadmill inclinations. The estimation strategy of the IMU placed on the thorax and the machine learning algorithm provided **much better** estimations of the duty factor, but with poor accuracy in extreme downhill conditions (i.e., -8)''. The authors tested only a single and not previously validated method for the estimation of initial and final contacts with IMUs on feet, thus it is preferable to only assess that the method proposed by the author for the estimation of the duty factor led to worse performances than the method using the IMU on the thorax. In addition, please provide the quantitative difference between the estimates of the two strategies.
7) I suggest to explicitly and clearly describe which IMU-based strategy performed better in the estimation of running parameters and indices, always reporting the quantitative differences.
8) Please stress better the limitations of the research, of the protocol and of the machine learning approach with the IMU on thorax.

Additional Materials

1) The additional materials is very verbose and could be shortened, especially in the first part dealing with the background, the rationale of the project, the description of the estimated metrics, and the datasets. Many information are already available in the paper and here are repeated.
2) The statistical analysis on the obtained results is also reported in the Results of the paper. Please insert only the figures and a brief description. Check the accordance between p-values reported in the Additional Materials and Results, which not always coincided.
3) *Validation dataset (10 random participants) – Stride frequency: running condition and estimated strategy*: *a*) this section deals with all the estimated parameters, not only the stride frequency; *b*) the Figure of the results on DFA across different running speeds shows the Optogait as second column, in contract with all the other similar figures, thus worsening the readability.
4) Please consider to change the measure unit '1/100' into '%'.
5) *Comparison between Movesense sensors at the foot level and Optogait* and *Comparison between neural network algorithm and Optogait*: In Bland-Altaman plots and figures showing the RMSE, please specify what the horizontal dashed lines stand for.

Reference

1. Cerone, G.L.; Nicola, R.; Caruso, M.; Rossanigo, R.; Cereatti, A.; Vieira, T.M. Running Speed Changes the Distribution of Excitation within the Biceps Femoris Muscle in 80 m Sprints. *Scand J Med Sci Sports* **2023**, doi:10.1111/sms.14341.
