# Supplementary material for: Indoor running temporal variability for different running speeds, treadmill inclinations, and three different estimation strategies
Source: PLoS One. 2023 Jul 20;18(7):e0287978. doi: 10.1371/journal.pone.0287978 (PMC10358961; doi:10.1371/journal.pone.0287978)
Supplement: Attachment — Submitted filename: rebuttal_R1.docx [file pone.0287978.s003.docx]

We would like to express our gratitude to the reviewers for taking the time to thoroughly review our manuscript. Their insightful feedback and comments demonstrate a remarkable level of expertise, dedication, and attention to details. We are motivated to enhance our work based on their comments, as we believe that, if each point is carefully considered and addressed, the overall quality of the manuscript will improve.

After thoroughly reviewing the manuscript and taking into consideration the valuable feedback provided by the reviewers, we have made the decision to enhance our analysis by designing an additional model that addresses the estimation of the single DFA-alpha variability index. We believe that this addition will significantly contribute to the comprehensiveness and overall quality of our work. Although this specific model was not originally included in the initial version of the manuscript, we sincerely hope that the reviewers will acknowledge and appreciate our efforts to provide a more comprehensive study. We are confident that this addition will strengthen the scientific contributions of our research and address the reviewers' concerns effectively.

**Reviewer 1**

**Reviewer #1.0**: The study is interesting and worthy of investigation. I would congratulate the authors as this work was the most reproducible and transparent work that I have reviewed, as all the codes and analysis are in repositories. My major comment is regarding the results section, when there is a lack of data to understand the results without the necessity to check the supplementary material (see specific comment below).

Thank you for your kind words about the manuscript and the overall project. We strongly value open science and the opportunities that comes from sharing codes and the data.

**Reviewer #1.1**: Introduction, end of the first paragraph: Please, include one or two sentences describing both parameters and the differences between them.

Thank you for your input. Additional background is now provided about DFA and Higuchi's D in the introduction. "Particularly, DFA-alpha is a measure of the correlation properties of a signal, and it is related to the size of fluctuations in signal changes with the length of time over which the fluctuations are measured. Higuchi's D is a measure of the fractal dimension of a signal, which is a way of characterizing its overall complexity."

**Reviewer #1.2**: Methods, “age 25 (6); BMI 20.76 (1.75)”: Please, include the units of both variables. Moreover, I suggest avoiding the abbreviation of males and females.

Done, thank you.

**Reviewer #1.3**: Statistical analysis: I recommend including the effect size analysis to improve the interpretation of the results.

Thank you for your comment. Effect size calculation has been included in the new version of the manuscript for the ANOVA tests.

**Reviewer #1.4**: Results: Even if all the results are published in one report, this is not entirely easy for readers. I think that the authors should add some figures in the results section to show the data obtained more visually so that the values can be viewed comfortably. Currently, as the results section is, it is known in what they exist and not significant differences, but without accessing said report you cannot see the values, which is not valid. The article must contain all the essential information by itself without having to check the supplementary material.

This is in line with comments **R2.25-29**. Please consider that we included relevant tables and plots in the new version of the manuscript. You can still find useful information in the Additional Material.

**Reviewer #1.5**: Results, “-8 vs +2 (p=0.00237) and +5 (p=0.00106) and +8 (p=0.00749), -5 vs +2 (p=0.0087) and +5 (p=0.00467) and +8 (p=0.02), -2 vs +2 (p=0.02) and +5 (p=0.01).”: I recommend including the DFA-α values in the parenthesis to understand the differences between inclinations or include a figure with the data.

We agree with this comment. Figures and tables with the data have been included in the new version of the manuscript.

**Reviewer #1.6**: Discussion, first paragraph: I recommend including the main results observed by the study.

Thank you. A brief synopsis of the results has been included in the new version of the manuscript. "The results suggest that using both IMU configurations is effective for estimating stride frequency and duty factor with good accuracy. Using a single IMU on the thorax together with a machine learning algorithm resulted in more accurate estimates of duty factor than the IMU on the feet. However, caution is advised when measuring running variability indices, especially in extreme downhill conditions."

**Reviewer #1.7**: Discussion: include at the end a paragraph about the limitations of the study.

This is in line with **R2.24**. We agree with your comment. Limitations of the study were not discussed with enough detail. Please notice that now the manuscript has been improved.

**Reviewer 2**

**Reviewer #2.0**: The paper analyses the results obtained in terms of stride frequency, duty factor, and detrended fluctuation analysis and Higuchi’s D index of the stride frequency obtained during indoor running at different speeds and treadmill inclinations. In general, the paper has a high-quality English language, but the overall structure must be improved, the results must be more elaborated, and the discussion must be clearer. The authors declared to accomplish two aims: i) provide normative values, ii) compare three different strategies. However, the first aim is only discussed in the additional materials. In the Results the authors should revise the reported values and sentences. I would recommend accepting the manuscript only if the following points will be addressed.

Thank you for taking the time to produce this valuable review. We agree with what you state, and we believe that now a better version of the manuscript has been produced.

**Reviewer #2.1**: I would not mention ‘spatio-temporal variability’ since the only two estimated running parameters are the stride frequency and the duty factor, which are both only temporal.

Thank you for your comment. We agree with what you suggested; therefore, we used the term temporal variability in the new version of the manuscript.

**Reviewer #2.2**: Are 20 subjects sufficient to provide normative values of the estimated quantities? The authors should clarify that.

We agree wholeheartedly with you. It seems a big stretch to try to infer "normative values" from such a small sample. This is linked to other comments (**R2.3** and **R2.7**).

**Reviewer #2.3**: According to the previous points, I suggest changing the title in ‘Indoor running temporal variability for different running speeds, treadmill inclinations, and three different estimation strategies’.

We agree with the point raised. We are going to change the title accordingly to your suggestions (**R2.2** and **R2.3**).

**Abstract**

**Reviewer #2.4**: The authors mention the accuracy of the machine learning algorithm using the IMU on the thorax in the estimation of the duty factor, but the paper is focused on the estimation of the variability indices. I suggest to briefly mention the main results obtained in the estimation of the detrended fluctuation analysis and Higuchi’s D index, specifying which of them is more accurately estimated with the IMU on the thorax or on the feet.

Thank you. The abstract has been modified accordingly.

**Introduction**

**Reviewer #2.5**: The introduction of the variability indices is discussed nicely. Although, the introduction of the estimated temporal parameters is quite missing. The information required to estimate the stride frequency and the duty factor are the running events (initial and final contact instants). I do recommend citing some studies assessing running events, stride frequency, and duty factor using different technologies.

Thank you for your suggestion. An up-to-date systematic review of the literature on the topic has been included in the new version of the manuscript. Mason, R., Pearson, L.T., Barry, G. et al. Wearables for Running Gait Analysis: A Systematic Review. Sports Med 53, 241–268 (2023). https://doi.org/10.1007/s40279-022-01760-6. Furthermore, the review by Oeveren et al. 2021 was also already cited together with the review by Vernillo et al. 2016. Given that temporal running kinematic variables are quite common in the scientific literature, we think that the level of information provided is sufficient. We would be glad to include additional information if you think that this point is still not sufficiently addressed.

**Reviewer #2.6**: The authors mentioned some technologies confined in the laboratory setting in the following clause: ‘‘Running variability is indeed best assessed in laboratory conditions, where optoelectronic devices, force platforms, motion capture systems, or pressure insoles are available’’. Although, pressure insoles are wearable and portable. A study estimating the running cycle (stride) durations in-the-field with pressure insoles is discussed in a recent paper: https://doi.org/10.1111/sms.14341. Thus, I do recommend to explicitly mention that running temporal parameters can be assessed in laboratory conditions using motion capture systems, force platforms, or instrumented treadmills, while, in outdoor conditions, IMUs are typically the best alternative, since they can provide the kinematic of the body segments where they are attached, and pressure insoles can be a portable reference for temporal parameters [1].

We agree that pressure insoles can be considered "portable” but stating that they can be used as "wearables" with the same simplicity and light portability of IMUs is a bit of a stretch in our opinion. There is no doubt that, in the future, we might see pressure insoles to be seamlessly integrated in the footwear design, but this is not the case for the present. We do agree, however, that information that might come from the pressure insoles can be key in the calculation/estimation of the temporal parameters in the outdoors. The reference cited in **R2.30** (https://doi.org/10.1111/sms.14341) will be included in the new version of the manuscript as it is pertinent and provides a great up-to-date example about how pressure insoles can be used in outdoor conditions.

**Reviewer #2.7**: The reference values are reported only in the additional materials and are assessed only on 20 runners. I suggest mentioning a single general aim of the study: the estimation of stride frequency, duty factor, and associated variability using three different strategies.

Thank you for your comment. This is in line with **R2.2-2.3**.

**Methods**

**Reviewer #2.8**: Normative values (Optogait): Were the DFA-α and Higuchi’s D values calculated for both stride frequency and duty factor? It seems that they were calculated only on a single parameter (stride frequency). The authors should clarify that.

Thank you, very good point. We included a comment in the Methods.

**Reviewer #2.9**: Other estimation strategies (IMU on the feet): The description of the method with IMU on feet should be improved. The authors mentioned a reference study, but they modified it after a trial and error approach. Thus, it is preferable to explicitly describe how the initial and final contacts were found, since they are crucial for the calculation of stride frequency and duty factor. A reference to Figure 1 can be added.

We acknowledge that the previous version of the manuscript might have been unclear. We want to highlight that taking the norm of the accelerations was one of the alternatives discussed by the authors of the referenced manuscript (Falbriard 2018). However, Falbriard et al. 2018 did not report that this method was the best one compared to all others they investigated. However, based on our own experience and after trial and error, we chose to use this methodology because it has proven to be more reliable and robust for our particular application. Conducting an extended and rigorous validation of the methods proposed and discussed by Falbriard et al. 2018 was outside the scope of this research. The new version of the manuscript includes additional information on this topic. Please notice that this is of interest for the next comment (**R2.10**).

**Reviewer #2.10**: Figure 1: Which inertial signals are shown? Please show the acceleration norm (which is used to estimate the running events from the feet) and highlight not only the initial contact instants but also the final contact instants in the figure.

We are sorry to hear that the use of the inertial signals in the image may have generated some confusion. We included the signals only for illustrative purposes, as providing detailed information about the signals and the detection of running cycles and variables would require a level of detail that is not appropriate within the context of this figure. We understand that providing more information would not be proportionate with other aspects of the process depicted in the same figure. However, we are open to adjusting the figure if you think it's strictly necessary and we appreciate your comment and feedback on this aspect of our work. Please notice that this is in line with the previous comment (**R2.9**).

**Results**

**Reviewer #2.11**: Please add the explicit information of the result of the normality test for each tested distribution.

Thank you for this comment. This has been really useful for us to check our results carefully. We realised that the normality conditions were checked for the entire sample but, in some cases, not for the single groups. After checking the literature, we found that this approach might be more rubust. Therefore, the results of the normality tests are reported for every group (running condition) for every variable (i.e., stride frequency, duty factor, DFA-alpha and Higuchi's D). In case this normality assumption was violated (it happened in few cases), a non-parametric version of the ANOVA and t-test were conducted. Please also check comment **R2.26**.

**Reviewer #2.12**: Reference and estimated quantities are only reported in the Additional Materials. In the Results section only the statistical analysis is described. Please add a table or a brief description of the estimates of stride frequency, duty factor, variability indices, and their errors according to the different running conditions. It will increase the readability of the paper and the understanding of the statistical analysis.

Thank you for your comment. A table which includes the values of stride frequency, duty factor and variability indices assessed with the gold-standard optical system has been included.

**Reviewer #2.13**: Normative values: The following sentence is reported: ‘‘ANOVA conducted on running frequency with different treadmill inclinations returned p=0.1322, so the running frequencies are dependent from treadmill inclination’’. Please revise the results of the statistical analysis, in particular the above one.

Thank you for your comment. The manuscript has been amended.

**Reviewer #2.14**: Other estimation strategies: The following sentences are reported: ‘‘The p-value for the interaction between running condition and estimation strategy is 0.9052, which indicates that the stride frequency value does not depend on the estimation strategy’’ and ‘‘The p-value for the interaction between running condition and estimation strategy is 0.9998, which indicates that the stride frequency value does not depend on the estimation strategy’’. Please revise and rephrase those sentences. Can ‘‘the p-value for the interaction between running condition and estimation strategy is XXX, which indicates that there is no interaction between running speed/treadmill inclination and estimation strategy’’ be more suitable?

Thank you for your comment. Yes, this sounds suitable, and your suggestion will be indeed directly included in the new version of the manuscript.

**Reviewer #2.15**: Sometimes the authors refer to the significant differences between the estimations with the IMU on the feet/thorax and the other two estimation strategies. These kinds of information are preferable to be inserted in the Discussion, instead of the Results. Furthermore, the mentioned quantities and errors are only reported in the Additional Materials. I suggest to explicitly mention the values of the obtained errors to help the reader to understand if some differences are significant but not relevant or vice versa.

Thanks. Comments about the significant differences between the estimation strategies were deleted from the Results.

**Reviewer #2.16**: Please revise the following sentence ‘‘Two-way ANOVA conducted on Higuchi’s D with different treadmill inclinations returned p=0.364 for the treadmill inclination factor and p<0.0001 for the estimation strategy, so the hypothesis that all duty factor values are equal for different estimation strategies is rejected. This is due to the significant differences between the estimations with the IMU on the feet and the two other estimations strategies’’. It seems to be not in accordance with the results reported in the Additional Materials (which highlighted that the IMU on the thorax worsened the results) and to include some typing errors.

Thank you so much for pointing this out. Indeed, the p<0.0001 returned by the ANOVA is mostly due (as presented in the figures) by the differences between the estimation strategy that makes use of the IMU on the thorax + machine learning model. Figures highlighting statistical differences between estimation strategies have now been included in the manuscript.

**Discussion**

**Reviewer #2.17**: Is the goal to present normative values for indoor running kinematics and variability achieved? I suggest to remove this first aim, as explained before.

This is in line with previous comments: **R2.2**, **R2.3**, and **R2.7**. Yes, as previously mentioned, we fully agree with you on this point. We are going to remove this aim and to adjust the manuscript.

**Reviewer #2.18**: The comparison with the results in the literature of interest in terms of DFA-α can be improved. If you declare to provide normative values, it might be crucial the comparison with other refence values in the literature. What quantitative normative values are reported in the literature? Which are the studies reporting normative values similar to the ones obtained by the authors? Please report the quantitative differences between the values obtained by the authors and other studies in the literature. It can helpful to know the ranges to understand how much they overlap.

According to other comments (e.g., **R2.2**, **R2.3**, **R2.7, and R2.17**) the values reported in this manuscript cannot be regarded as "normative values". We included some additional comparisons with other values reported in the literature (see also **R2.21**). We think that the relevant literature on the topic was already mentioned in the previous version of the manuscript, but the numerical values were missing. We are going to integrate the Discussion with data from similar studies.

**Reviewer #2.19**: The comparison with the results in the literature of interest in terms of stride frequency and duty factor estimated from IMUs on feet or thorax is missing. Are the obtained errors comparable with similar studies? In addition, see the consideration raised above for DFA- α.

Thank you for your comment. Additional information regarding a comparison with the literature for the estimation of the duty factor with machine learning models and IMU-based methodologies has been included.

**Reviewer #2.20**: The comparison with the results in the literature of interest in terms of Higuchi’s D index is missing. Are there any studies estimating this variability index in running? Are the results similar? In addition, see the consideration raised above for DFA- α.

We agree entirely with this comment. This relates also with the previous comments. In the previous version of the manuscript this aspect was poorly addressed. We hope you will find that the new version of the manuscript includes more detail about the values of the Higuchi's D in the context of the existing literature.

**Reviewer #2.21**: Are there any other studies investigating variability varying speed and/or surface inclination? Please cite them. The findings of statistical analysis is in accordance with the literature in terms of the observed significant (or not) dependences of the variability indices on running conditions?

Unfortunately, we are not aware on any study conducted on different treadmill inclinations that evaluated running variability DFA-alpha or Higuchi's D. Therefore, our capacity to discuss our results and to put them in the context of the current literature is limited. A comment was included in the new version of the manuscript.

**Reviewer #2.22**: The following sentence is reported: ‘’In the estimation of the duty factor, the estimation strategy with the IMU on the feet was found to be not accurate across all the different running speeds and treadmill inclinations. The estimation strategy of the IMU placed on the thorax and the machine learning algorithm provided much better estimations of the duty factor, but with poor accuracy in extreme downhill conditions (i.e., -8)’’. The authors tested only a single and not previously validated method for the estimation of initial and final contacts with IMUs on feet, thus it is preferable to only assess that the method proposed by the author for the estimation of the duty factor led to worse performances than the method using the IMU on the thorax. In addition, please provide the quantitative difference between the estimates of the two strategies.

Thank you for your comment. We did not have the capacity to reproduce all the available methods for the calculation of the duty factor with an IMU placed on the feet of the runners. In this case, yes, we refer to the specific methodology that we implemented that, in our experience, was the most reliable. We did not report the values, or the results related to the trial-and-error process that led us to the conclusion that using the norm of the accelerations was the best choice for us.

However, to avoid any possible confusion, we amended the manuscript and we specified that when we refer to the method of the IMU placed on the feet of the runner, we specifically refer to our implementation of that methodology and we do not have the data to state that the methodology is better or worse in absolute terms. "It has to be highlighted that in this study a configuration where two IMUs were placed on each foot of the runner was not previously validated, so the results reported in this study might not be true for all the systems exploiting this sensor configuration."

**Reviewer #2.23**: I suggest to explicitly and clearly describe which IMU-based strategy performed better in the estimation of running parameters and indices, always reporting the quantitative differences.

This is related to **R2.22**.

**Reviewer #2.24**: Please stress better the limitations of the research, of the protocol and of the machine learning approach with the IMU on thorax.

Please consider that this is in line with **R1.7**. Limitations of the study are now presented and discussed in the new version of the manuscript.

**Additional Material**

**Reviewer #2.25**: The additional materials is very verbose and could be shortened, especially in the first part dealing with the background, the rationale of the project, the description of the estimated metrics, and the datasets. Many information are already available in the paper and here are repeated.

Please consider that **Rev#1** also raised some concerns about the structure and the verbose nature of the Additional Material in **R1.4**. We agree entirely. We shortened the Additional Material and tried to improve readability and overall flow.

**Reviewer #2.26**: The statistical analysis on the obtained results is also reported in the Results of the paper. Please insert only the figures and a brief description. Check the accordance between p-values reported in the Additional Materials and Results, which not always coincided.

We appreciate your thorough review of the Additional Material. We have also identified discrepancies in the reporting of certain p-values within the manuscript. We hope you will find the revised version of the Additional Material to be more effectively presented. Additionally, please be aware that after conducting group-specific tests for normality, some p-values have changed due to the implementation of non-parametric tests. Please notice that this is in line with your previous comment **R2.11**.

**Reviewer #2.27**: Validation dataset (10 random participants) – Stride frequency: running condition and estimated strategy: a) this section deals with all the estimated parameters, not only the stride frequency; b) the Figure of the results on DFA across different running speeds shows the Optogait as second column, in contract with all the other similar figures, thus worsening the readability.

Thank you so much for picking these errors up. Indeed, labels were inverted in the graph (your comment **R2.27b**).

**Reviewer #2.28**: Please consider changing the measure unit ‘1/100’ into ‘%’.

Thank you for picking this up. Done.

**Reviewer #2.29**: Comparison between Movesense sensors at the foot level and Optogait and Comparison between neural network algorithm and Optogait: In Bland-Altaman plots and figures showing the RMSE, please specify what the horizontal dashed lines stand for.

Thank you for the comment. The following information was included in the additional material: "It is common practice to include limits of agreement with horizontal dashed lines in Bland-Altman plots. These limits of agreement have been approximated with ±1.96 times the standard deviation of the difference between the gold standard and the estimated values. Additionally, horizontal lines corresponding to the selected minimum worthwhile difference were included."

**References**

**Reviewer #2.30**: 1. Cerone, G.L.; Nicola, R.; Caruso, M.; Rossanigo, R.; Cereatti, A.; Vieira, T.M. Running Speed Changes the Distribution of Excitation within the Biceps Femoris Muscle in 80 m Sprints. Scand J Med Sci Sports 2023, doi:10.1111/sms.14341.

The suggested citation was included in the manuscript.
